# Reconstruction algorithms for DNA-storage systems

**DOI:** 10.1038/s41598-024-51730-3

**Published:** 2024-01-23

**Authors:** Omer Sabary, Alexander Yucovich, Guy Shapira, Eitan Yaakobi

**Affiliations:** The Henry and Marilyn Taub Faculty of Computer Science, Technion, 3200003 Haifa, Israel

**Keywords:** Computational biology and bioinformatics, Data processing

## Abstract

Motivated by DNA storage systems, this work presents the *DNA reconstruction problem*, in which a length-*n* string, is passing through the *DNA-storage channel*, which introduces deletion, insertion and substitution errors. This channel generates multiple noisy copies of the transmitted string which are called *traces*. A *DNA reconstruction algorithm* is a mapping which receives *t* traces as an input and produces an estimation of the original string. The goal in the DNA reconstruction problem is to minimize the edit distance between the original string and the algorithm’s estimation. In this work, we present several new algorithms for this problem. Our algorithms look globally on the entire sequence of the traces and use dynamic programming algorithms, which are used for the *shortest common supersequence* and the *longest common subsequence* problems, in order to decode the original string. Our algorithms do not require any limitations on the input and the number of traces, and more than that, they perform well even for error probabilities as high as 0.27. The algorithms have been tested on simulated data, on data from previous DNA storage experiments, and on a new synthesized dataset, and are shown to outperform previous algorithms in reconstruction accuracy.

## Introduction

Over the past decade, significant advancements have been made in both DNA synthesis and sequencing technologies^1–7^. These advancements have also led to the emergence of DNA-based data storage technology. A DNA storage system consists of three main components. The first is the *DNA synthesis* which generates the *oligonucleotides*, also called *strands*, that encode the data. Due to the need for high throughput production with acceptable error rates, the length of these strands is typically limited to roughly 250 nucleotides^8^. The second part is a storage container with compartments which stores the DNA strands, however without order. Finally, *DNA sequencing* is performed to read back a representation of the strands, which are called *reads* or *traces*.

Current synthesis technologies are not able to generate a single copy for each DNA strand, but only multiple copies where the number of copies is in the order of thousands to millions. Moreover, sequencing of DNA strands is usually preceded by PCR amplification which replicates the strands^9^. Hence, every strand has multiple copies and several of them are read during sequencing.

The process of encoding the user’s binary data into DNA strands, as well as the reverse process of decoding, are external to the storage system. Both processes need to be designed to ensure the recoverability of the user’s binary data, even in the presence of errors. The decoding step consists of three main stages known as 1. *clustering*, 2. *reconstruction*, and finally 3. *error correction*. The clustering step is the process of partitioning the noisy sequencing reads into *clusters* based on their origin, i.e., each cluster contains noisy copies of the same original designed strand. Following the clustering step, the objective is to reconstruct each individual strand using all available noisy copies. This stage is the primary focus of study in this paper. Finally, any errors that remain after the reconstruction step, including mis-clustering errors, lost strands, and other error mechanisms, should be addressed using an error-correcting code.

During the second stage of the decoding, a reconstruction algorithm is performed on each cluster to retrieve the original strand from the noisy copies in the cluster. The availability of multiple copies for each strand enables error correction during this process. In fact, this setup falls within the broader framework of the *string reconstruction problem*, which involves recovering a string based on multiple noisy copies of it. Examples of these problems include the *sequence reconstruction problem*, initially investigated by Levenshtein^10,11^, as well as the *trace reconstruction problem*^12–16^. Generally, these models assume that information is transmitted through multiple channels, and the decoder, equipped with all channel estimations, exploits the inherent redundancy to correct errors.

Generally speaking, the main problem studied under the paradigm of the sequence reconstruction and trace reconstruction problems is to find the minimum number of channels that guarantee successful decoding either in the worst case or with high probability. However, in DNA-based storage systems we do not necessary have control on the number of strands in each cluster. Hence, the goal of this work is to propose efficient algorithms for the reconstruction problem as it is reflected in DNA-based storage systems where the cluster size is a given parameter. Then, the goal is to output a strand that is close to the original one so that the number of errors the error-correcting code should correct will be minimized. We present algorithms that work with a flexible number of copies and various deletion, insertion and substitution probabilities.

In our model we assume that the clustering step has been done successfully. This could be achieved by the use of indices in the strands and other advanced coding techniques; for more details see^17^ and references therein. Thus, the input to the algorithms is a cluster of noisy read strands, and the goal is to efficiently output the original strand or a close estimation to it with high probability. We also apply our algorithms on simulated data, on data from previously published DNA-storage experiments^18–20^, and on our new data. We compare the accuracy and the performance of our algorithms with state of the art algorithms known from the literature.

### DNA Storage and DNA Errors

In the last decade, several DNA storage experiments were conducted^20–23^. The processes of synthesizing, storing, sequencing, and handling DNA strands are susceptible to errors. Each step within these processes has the potential to introduce a notable number of errors independently. Furthermore, the DNA storage channel possesses distinct characteristics that set it apart from other storage media, such as tapes, hard disk drives, and flash memories. We will provide an overview of some of these dissimilarities and discuss the unique error behavior observed in DNA.

1. Deletion, insertion, and substitution errors can be introduced in both the read and synthesized strands during the processes of synthesis and sequencing.
2. Existing synthesis methods are incapable of producing a single copy for each strand. Instead, they generate thousands to millions of noisy copies, each with its own unique error distribution. Additionally, certain strands may have a substantially larger number of copies, while others may not have any copies at all.
3. The use of DNA for storage or other applications typically involves PCR amplification of the strands in the DNA pool^9^. The PCR process sometimes prefer some of the strands over others. Thus, it is possible that this process can effect the distribution of the number of copies of individual strands and their error profiles^24,25^.
4. Longer DNA strands can be sequenced using designated sequencing technologies, e.g. PacBio and Oxford Nanopore^20,23,26,27^. However, the error rates of these technologies can be higher, with deletions and substitutions as the most dominant errors^28,29^.
5. Furthermore, there are emerging synthesis technologies, e.g. photolithographic light-directed synthesis^28,30^, in which the reported error rates are up to 25%-30%.

A detailed characterization of the errors in the DNA-storage channel and more statistics about previous DNA-storage experiments can be found in^9,29^.

### This work

In this work, we introduce several novel reconstruction algorithms specifically tailored for DNA storage systems. Our primary goal is to address the reconstruction problem as it is reflected in DNA storage systems, and thus our algorithms focus on maximizing the similarity between the algorithms’ outputs and the original strands. Notably, our algorithms differ from previously published reconstruction algorithms in several key aspects. First, we do not impose any assumptions on the input. This means that the input can be arbitrary and does not necessarily adhere to an error-correcting code. Second, our algorithms are not restricted to specific cluster sizes, nor do they require dependencies between error probabilities or assume zero errors at specific strand locations. Third, we have the capability to control the complexity of our algorithms, allowing us to limit their runtime when applied to real data derived from prior DNA storage experiments. Finally, given that clusters in DNA storage systems can exhibit variations in size and error distributions, our algorithms are designed to minimize the edit distance between our output and the original strand, while considering that these errors can be corrected using an error-correcting code.

## Preliminaries and problem definition

We denote by $\Sigma _q =\{0,\ldots ,q-1\}$ the alphabet of size *q* and $\Sigma _q^* \triangleq \bigcup _{\ell =0}^\infty \Sigma _q^\ell$. The length of ${{\varvec{x}}}\in \Sigma ^n$ is denoted by $|{{\varvec{x}}}|=n$. The *Levenshtein distance* between two strings ${{\varvec{x}}},{{\varvec{y}}}\in \Sigma _q^*$, denoted by $d_L({{\varvec{x}}},{{\varvec{y}}})$, is the minimum number of insertions and deletions required to transform ${{\varvec{x}}}$ into ${{\varvec{y}}}$. The *edit distance* between two strings ${{\varvec{x}}},{{\varvec{y}}}\in \Sigma _q^*$, denoted by $d_e({{\varvec{x}}},{{\varvec{y}}})$, is the minimum number of insertions, deletions and substitutions required to transform ${{\varvec{x}}}$ into ${{\varvec{y}}}$, and $d_H({{\varvec{x}}},{{\varvec{y}}})$ denotes the *Hamming distance* between ${{\varvec{x}}}$ and ${{\varvec{y}}}$, when $|{{\varvec{x}}}| = |{{\varvec{y}}}|$. For a positive integer *n*, the set $\{1,\ldots ,n\}$ is denoted by [*n*].

The *trace reconstruction problem* was first proposed in^12^ and was later studied in several theoretical works; see e.g.^14–16,31^. Under this framework, a length-*n* string ${{\varvec{x}}}$, yields a collection of noisy copies, also called *traces*, ${{\varvec{y}}}_1, \ldots , {{\varvec{y}}}_t$ where each ${{\varvec{y}}}_i$ is independently obtained from ${{\varvec{x}}}$ by passing through a *deletion channel*, under which each symbol is independently deleted with some fixed probability $p_d$. Suppose the input string ${{\varvec{x}}}$ is arbitrary. In the trace reconstruction problem, the main goal is to determine the required minimum number of i.i.d traces in order to reconstruct ${{\varvec{x}}}$ with high probability. This problem has two variants. In the “worst case”, the success probability refers to all possible strings, and in the “average case” (“random case”) the success probability is guaranteed for a random (uniformly chosen) input string ${{\varvec{x}}}$.

The trace reconstruction problem can be extended to the model where each trace is a result of ${{\varvec{x}}}$ passing through a *insertion-deletion-substitution channel*. Here, in addition to deletions, each symbol can be switched with some substitution probability $p_{s}$, and for each *j*, with probability $p_i$, a symbol is inserted before the *j*th symbol of ${{\varvec{x}}}$. Under this setup, the goal is again to find the minimum number of channels which guarantee successful reconstruction of ${{\varvec{x}}}$ with high probability. For this purpose, it should be noted that there are many interpretations for the insertion-deletion-substitution, most of them differ on the event when more than one error occured on the same index. Our interpretation of this channel is described in “Results” section.

Motivated by the storage channel of DNA and in particular the fact that different clusters can be of different sizes, this work is focused on another variation of the trace reconstruction problem, which is referred by the *DNA reconstruction problem*. The setup is similar to the trace reconstruction problem. A length-*n* string ${{\varvec{x}}}$ is transmitted *t* times over the *deletion-insertion-substitution channel* and generates *t* traces ${{\varvec{y}}}_1, {{\varvec{y}}}_2, \ldots , {{\varvec{y}}}_t$. A *DNA reconstruction algorithm* is a mapping $R: (\Sigma _q^*)^t \rightarrow \Sigma _q^*$ which receives the *t* traces ${{\varvec{y}}}_1, {{\varvec{y}}}_2, \ldots , {{\varvec{y}}}_t$ as an input and produces $\widehat{{{\varvec{x}}}}$, an estimation of ${{\varvec{x}}}$. The goal in the DNA reconstruction problem is to minimize $d_e({{\varvec{x}}},\widehat{{{\varvec{x}}}})$, i.e., the edit distance between the original string and the algorithm’s estimation. When the channel of the problem is the *deletion channel*, the problem is referred by the *deletion DNA reconstruction problem* and the goal is to minimize the Levenshtein distance $d_L({{\varvec{x}}},\widehat{{{\varvec{x}}}})$. While the main figure of merit in these two problems is the edit/Levenshtein distance, we will also be concerned with the complexity, that is, the running time of the proposed algorithms. Due to lack of space, this paper presents only the main results for the DNA reconstruction problem. Our algorithm and results of the deletion DNA reconstruction problem can be found in the supplementary material.

## Related work

This section reviews the related works on the different reconstruction problems. In particular we list the reconstruction algorithms that have been used in previous DNA storage experiments. A summary of some of the main theoretical results on the trace reconstruction problem can be found in the supplementary material.

Batu et al.^12^ studied the trace reconstruction problem as an abstraction and a simplification of the multiple sequence alignment problem in bioinformatics. Here the goal is to reconstruct the DNA of a common ancestor of several organisms using the genetic sequences of those organisms. They focused on the deletion case of this problem and suggested a majority-based algorithm to reconstruct the sequence, which they referred by the *bitwise majority alignment* (*BMA*) *algorithm*. They aligned all traces by considering the majority vote per symbol from all traces, while maintaining pointers for each of the traces. If a certain symbol from one (or more) of the traces does not agree with the majority symbol, its pointer is not incremented and it is considered as a deletion. They showed and proved that even though this technique works locally for each symbol, its success probability is relatively high when the deletion probability is small enough.

Viswanathan and Swaminathan presented in^32^ a BMA-based algorithm for the trace reconstruction problem under the deletion-insertion-substitution channel. Their algorithm extends the BMA algorithm so it can support also insertions and substitutions. It works iteratively on “segments” from the traces, where a segment consists of consecutive bits and its size is a fixed fraction of the trace that is given as a parameter to the algorithm.

Gopalan et al.^33^ used the approach of the BMA algorithm from^12^ and extended to support DNA storage systems. In their approach, they considered deletion errors as was done in^12^, and also considered insertion and substitution errors. Their method works as follows. They considered a majority vote per symbol as was done in^12^, but with the following improvements. For any trace that its current symbol did not match the majority symbol, they used a “lookahead window” to look on the next 2 (or more) symbols. Then, they compared the next symbols to the majority symbols and classified it as an error accordingly. Organick et al. conducted a large scale DNA storage experiments in^20^ where they successfully reconstructed their sequences using the reconstruction algorithm of Gopalan et al.^33^.

For the case of sequencing via nanopore technology, Duda et al.^34^ studied the trace reconstruction problem, while considering insertions, deletions, and substitutions. They focused on dividing the sequence into homopolymers (consecutive replicas of the same symbol), and proved that the number of copies required for accurately reconstructing a long strand is logarithmic with the strand’s length. Yazdi et al. used in^23^ a similar but different approach in their DNA storage experiment. They first aligned all the strands in the cluster using the multiple sequence alignment algorithm MUSCLE^35,36^. Then, they divided each strand into homopolymers and performed majority vote to determine the length of each homopolymer separately. Their strands were designed to be balanced in their GC-content, which means that 50% of the symbols in each strands were G or C. Hence, they could perform additional majority iterations on the homopolymers’ lengths until the majority sequence was balanced in its GC-content. All of these properties guaranteed successful reconstruction of the strands and therefore they did not need to use any error-correcting code in their experiment^23^. Another related characterization of the DNA storage channel was discussed in^37^. In their work, the error model is different compared to our work, since it also includes rearrangement errors. In rearrangement errors several parts of two or more strands are stuck to each other to inform one read. The suggested reconstruction method of their work was based on de Bruijn graphs.

For the case where the error probabilities are priorly known, Bar-Lev et al.^38^ and Srinivasavaradhan et al.^39^ presented two different methods to solve the reconstruction problem. Srinivasavaradhan et al. based their method on trellises that model the probability to obtain the traces from any possible sequence, and then returning the sequence with the highest probability. Bar-Lev et al. presented a DNN based algorithm that is trained with labeled simulated data and then uses transformer to estimate the original sequence from its noisy copies.

## Methods

This section studies the DNA reconstruction problem. Assume that a cluster consists of *t* traces, ${{\varvec{y}}}_1, {{\varvec{y}}}_2, \ldots , {{\varvec{y}}}_t$, where all of them are noisy copies of a synthesized strand. This model assumes that every trace is a sequence that is independently received by the transmission of a length-*n* sequence ${{\varvec{x}}}$ (the synthesized strand) through a deletion-insertion-substitution channel with some fixed probability $p_d$ for deletion, $p_i$ for insertion, and $p_s$ for substitution. Our goal is to propose an efficient algorithm which returns $\widehat{{{\varvec{x}}}}$, an estimation of the transmitted sequence ${{\varvec{x}}}$, with the intention of minimizing the edit distance between ${{\varvec{x}}}$ and $\widehat{{{\varvec{x}}}}$. In our simulations, we consider several values of *t* and a wide range of error probabilities. Additionally, we tested our suggested algorithm on new biological data sets, as well as on data from previous DNA storage experiments^18–20,28,39^; see more details in “Results” section. Due to space limitation, some of our algorithms, as well as more simulations and examples can be found in the supplementary material.

### Definitions and notations

While all previous works of reconstructing algorithms used variations of the majority algorithm on localized areas of the traces, we take a different global approach to tackle this problem. Namely, the algorithms presented in this work are heavily based on the *maximum likelihood decoder* for multiple deletion channels as studied recently in^40,41^ as well as the concepts of the *shortest common supersequence* and the *longest common subsequence*. Hence, we first briefly review the main ideas of these concepts, while the reader is referred to^40^ for a more comprehensive study of this summary.

For a sequence ${{\varvec{x}}}\in \Sigma _q^*$ and a set of indices $I\subseteq [|{{\varvec{x}}}|]$, the sequence ${{\varvec{x}}}_I$ is the *projection* of ${{\varvec{x}}}$ on the indices of *I* which is the subsequence of ${{\varvec{x}}}$ received by the symbols at the entries of *I*. A sequence ${{\varvec{x}}}\in \Sigma ^*$ is called a *supersequence* of ${{\varvec{y}}}\in \Sigma ^*$, if ${{\varvec{y}}}$ can be obtained by deleting symbols from ${{\varvec{x}}}$, that is, there exists a set of indices $I\subseteq [|{{\varvec{x}}}|]$ such that ${{\varvec{y}}}= {{\varvec{x}}}_I$. In this case, it is also said that ${{\varvec{y}}}$ is a *subsequence* of ${{\varvec{x}}}$. Furthermore, ${{\varvec{x}}}$ is called a *common supersequence* (*subsequence*) of some sequences ${{\varvec{y}}}_1,\ldots ,{{\varvec{y}}}_t$ if ${{\varvec{x}}}$ is a supersequence (subsequence) of each one of these *t* sequences. The *length of the shortest common supersequence* (*SCS)* of ${{\varvec{y}}}_1,\dots ,{{\varvec{y}}}_t$ is denoted by $\textsf{SCS}({{\varvec{y}}}_1,\dots ,{{\varvec{y}}}_t)$. The set of all shortest common supersequences of ${{\varvec{y}}}_1,\ldots ,{{\varvec{y}}}_t\in \Sigma ^*$ is denoted by ${{\mathcal {S}}}\textsf{CS}({{\varvec{y}}}_1,\ldots ,{{\varvec{y}}}_t)$. Similarly, the *length of the longest common subsequence* (*LCS)* of ${{\varvec{y}}}_1,\dots ,{{\varvec{y}}}_t$, is denoted by $\textsf{LCS}({{\varvec{y}}}_1,\dots ,{{\varvec{y}}}_t)$, and the set of all longest subsequences of ${{\varvec{y}}}_1,\dots ,{{\varvec{y}}}_t$ is denoted by ${{\mathcal {L}}}\textsf{CS}({{\varvec{y}}}_1,\ldots ,{{\varvec{y}}}_t)$.

Before we present the algorithm, we list here several more notations and definitions. An *error vector* of ${{\varvec{y}}}$ and ${{\varvec{x}}}$, denoted by $EV({{\varvec{y}}}, {{\varvec{x}}})$, is a vector of minimum number of edit operations to transform ${{\varvec{y}}}$ to ${{\varvec{x}}}$. Each entry in $EV({{\varvec{y}}}, {{\varvec{x}}})$ consists of the index in ${{\varvec{y}}}$, the original symbol in this index, the edit operation and in case the operation is an insertion, substitution the entry also includes the inserted, substituted symbol, respectively. Note that for two sequences ${{\varvec{y}}}$ and ${{\varvec{x}}}$, there could be more than one sequence of edit operations to transform ${{\varvec{y}}}$ to ${{\varvec{x}}}$. The edit distance between a pair of sequences is computed using a dynamic programming table and the error vector is computed by backtracking on this table. Hence, $EV({{\varvec{y}}}, {{\varvec{x}}})$ is not unique and can be defined uniquely by giving priorities to the different operation in case of ambiguity. That is, if there is an entry in the vector $EV({{\varvec{y}}}, {{\varvec{x}}})$ (from the last entry to the first), where more than one edit operation can be selected, then, the operation is selected according to these given priorities. The error vector $EV({{\varvec{y}}}, {{\varvec{x}}})$ also maps each symbol in ${{\varvec{y}}}$ to a symbol in ${{\varvec{x}}}$ (and vice versa). We denote this mapping as $V_{EV}({{\varvec{y}}}, {{\varvec{x}}}): \{1,2,\ldots , |{{\varvec{y}}}| \} \rightarrow \{1,2, \ldots , |{{\varvec{x}}}|\} \cup \{ ? \}$, where $V_{EV}({{\varvec{y}}}, {{\varvec{x}}})(i)=j$ if and only if the *i*th symbol in ${{\varvec{y}}}$ appears as the *j*th symbol in ${{\varvec{x}}}$, with respect to the error vector $EV({{\varvec{y}}}, {{\varvec{x}}})$. Note that in the case where the *i*th symbol in ${{\varvec{y}}}$ was classified as a deleted symbol in $EV({{\varvec{y}}}, {{\varvec{x}}})$, $V_{EV}({{\varvec{y}}}, {{\varvec{x}}})(i)= ?$. Furthermore, since inserted symbols appear only in ${{\varvec{x}}}$ and not in ${{\varvec{y}}}$, these symbols are not represented in the mapping $V_{EV}({{\varvec{y}}}, {{\varvec{x}}})$, and are only referred as insertions in the error vector $EV({{\varvec{y}}}, {{\varvec{x}}})$. The mapping $V_{EV}({{\varvec{y}}}, {{\varvec{x}}})$ can also be represented as a vector of size $|{{\varvec{y}}}|$, where the *i*th entry in this vector is $V_{EV}({{\varvec{y}}}, {{\varvec{x}}})(i)$. The *reversed cluster* of a cluster ${{\textbf{C}}}$, denoted by ${{\textbf{C}}}^R$, consists of all the traces in ${{\textbf{C}}}$ where each one of them is reversed. The reverse trace of ${{\varvec{y}}}_i \in {{\textbf{C}}}$ is denoted by ${{\varvec{y}}}_i^R\in {{\textbf{C}}}$. The symbols of each trace ${{\varvec{y}}}_i^R \in {{\textbf{C}}}^R$ are arranged in reverse order compared to how they appear in their original form ${{\varvec{y}}}_i \in {{\textbf{C}}}$. For example, for the cluster ${{\textbf{C}}}= \{ {{\varvec{y}}}_1 = ACGTC, {{\varvec{y}}}_2 = CCGTA \}$, the reversed cluster is ${{\textbf{C}}}^R = \{ {{\varvec{y}}}_1^R = CTGCA, {{\varvec{y}}}_2^R = ATGCC \}$.

### The iterative reconstruction algorithm

We are now ready to discuss the main algorithm of this work, referred to as the *iterative reconstruction algorithm* or in short *ITR algorithm*. The ITR algorithm receives the design length *n* and a cluster of *t* noisy traces, all generated from the same design strand ${{\varvec{x}}}$. The algorithm consists of several different procedures that are used to create *candidates* from the traces. Candidates are revised version of traces from the cluster, or some other estimations that are created by the algorithm and its procedures. The revisions applied on a trace are intended to decrease the edit distance between the resulting candidate and the designed sequence ${{\varvec{x}}}$. The candidates that are being created by the algorithm’s procedures are then assessed by the algorithm, and a score is given to each of them based on their number of occureneces and their average edit distance to the other traces in the cluster. Finally, the candidate with the highest score is returned by the algorithm (while ties are broken arbitrarily).

The different procedures that are used in the algorithm are given below in a high level description. For a detailed description of each of the procedures, as well as the ITR algorithm, the reader is referred to the supplementary material of this paper.

1. **The Error-Vector Majority Algorithm (EV Algorithm).**
  **Brief description.** The EV algorithm selects a random trace from the cluster ${{\varvec{y}}}_k$ and uses dynamic programming in a pairwise manner to compute the required vector of edit operations (insertion, deletion, or substitution) that should be taken in order to transform ${{\varvec{y}}}_k$ into any of the other traces in the cluster. Then, the algorithm calculates the most common operation on each of the symbols, and if it finds out that they include insertions or substitutions, it performs these operations on ${{\varvec{y}}}_k$. Deletions are ignored by the EV algorithm, since in most of the cases they are corrected more accurately by the PP algorithm, which is described next. The revised version of ${{\varvec{y}}}_k$ is considered as a candidate.
  **Intuition and motivation.** This algorithm extends the majority vote approach that was used in the BMA algorithm^12^ and in the BMA lookahead^33^. By using dynamic programming, the algorithm takes into account that deletion, insertion, and substitution errors occur. Thus, it is possible to detect specific symbols that were inserted into ${{\varvec{y}}}_k$ and do not appear in most of the other traces, or symbols that were substituted with others. Therefore, these two types of errors are corrected by the EV algorithm. The correction of deleted symbols is done by the PP algorithm which is described next.
  **Computational Complexity.** The algorithm iterates on every pairing of ${{\varvec{y}}}_k$ with another trace ${{\varvec{y}}}_i$, for $i\ne t$, where the cluster size is *t*, thus it works on $(t-1)$ pairs. For each such pairs of traces, the algorithm uses dynamic programming to calculate the edit distance, which works in quadratic complexity with the design length *n*. Thus, the complexity of this algorithm is ${{\mathcal {O}}}(tn^2)$.
2. **The Pattern Path Algorithm (PP Algorithm).**
  **Brief description.** The PP algorithm explores *patterns* in the cluster, which are subsequences that appear around the same location in two or more traces. To do so, the algorithm starts with a trace from the cluster ${{\varvec{y}}}_k$, and uses dynamic programming in order to find the longest common subsequence (LCS) of ${{\varvec{y}}}_k$ and any of the other traces in the cluster. These LCSs are then used to create a directed acyclic graph that describes the different patterns, and their location together with their occurrences in the computed LCSs. The longest path in this graph induces a sequence that is then serves as a candidate.
  **Intuition and motivation.** This algorithm exploits the LCS of ${{\varvec{y}}}_k$ and any of the other traces in the cluster to detect the most common patterns in the cluster. The graph is used to represent the different patterns, their location and the number of times they appeared in each of the observed LCSs. The weight of the edges in the graph are defined by the number of times each pattern was seen in a specific location in the LCSs. Therefore, the longest path in the graph, is served as the algorithm selected candidate. The patterns that are computed by the PP algorithm also consider deletions. The pattern of a deleted symbol includes not just the deleted symbol itself but also the two symbols preceding it in the traces and the two symbols immediately following it. Moreover, since the patterns in the graph are weighted by their frequency of occurrence, we believe that the PP algorithm is able to correct deletion errors more precisely. Thus, the PP algorithm is used by the VR algorithm and the HR algorithm (described below) to correct deletion errors.
  **Computational Complexity.** This algorithm calculates the LCS of ${{\varvec{y}}}_k$ and each of the other traces in the cluster. The cluster size is *t*, and thus the algorithm calculates the LCS for $t-1$ pairs. The calculation of the LCS is done with dynamic programming which takes ${{\mathcal {O}}}(n^2)$ complexity. Then, for every such a pair, the algorithm calculates the patterns that were observed in the LCS and this process is done in linear time with the length. Lastly, the algorithm creates the directed acyclic graph, in which it has at most *nt* vertices. Hence, the complexity of finding its longest path is linear with respect to the number of vertices and number of edges, which is at most $nt^2$. To conclude, the overall complexity of this algorithm is ${{\mathcal {O}}}(n^2t+nt^2)$.
3. **The Vertical Reconstruction Algorithm (VR Algorithm).**
  **Brief description.** The VR algorithm is invoked on each of the traces in the cluster. In each of these iterations, the VR algorithm starts with the *k*th trace in the cluster, ${{\varvec{y}}}_k$, and performs the following algorithms on it. First, the algorithm performs the EV algorithm to correct substitution and insertion errors in ${{\varvec{y}}}_k$ and the PP Algorithm to correct deletions in ${{\varvec{y}}}_k$. The correction of these errors is done by editing ${{\varvec{y}}}_k$ based on the algorithms’ outputs. The revised version of ${{\varvec{y}}}_k$ is then inserted into the cluster. The algorithm is invoked again on the updated cluster, with the new inserted revised version of ${{\varvec{y}}}_k$.
  **Intuition and motivation.** This algorithm is invoked twice in two iterations on each trace in the cluster ${{\varvec{y}}}_k$ (for $1 \leqslant k \leqslant t$). In the first iteration, it is performed on the original cluster, and in the second time it is invoked on the updated cluster that include the traces of the cluster with one additional trace, which is the algorithm estimation from the first iteration. This algorithm employs the PP and EV algorithms on ${{\varvec{y}}}_k$ to edit it with the goal of correcting any of its errors. Then, the revised version of ${{\varvec{y}}}_k$ is inserted into the cluster, and the algorithm is invoked one additional time on the updated cluster.
  **Computational Complexity.** For any trace ${{\varvec{y}}}_k \in {{\textbf{C}}}$, the VR algorithm works in two iterations, in which the EV algorithm, the PP algorithm are performed two, four times, respectively. Thus, its complexity for a single trace is given by $6(n^2t+nt^2) = {{\mathcal {O}}}(n^2t+nt^2)$, and its total complexity is ${{\mathcal {O}}}(n^2t^2)$.
4. **The Horizontal Reconstruction (HR Algorithm).**
  **Brief description.** Similarly to the VR algorithm, the HR algorithm performs the EV algorithm to correct substitution and insertion errors in ${{\varvec{y}}}_k$ and the PP Algorithm to correct deletions in ${{\varvec{y}}}_k$, for any ${{\varvec{y}}}_k \in {{\textbf{C}}}$. However, this algorithm creates the set of candidate differently. In the HR algorithm, the cluster is updated with the addition of the revised versions of ${{\varvec{y}}}_k$ after any iteration of the EV/PP algorithm (where in the VR algorithm, the cluster is updated only after performing both of them). That is, any iteration of each of these algorithms is followed by considering a new candidate, which is the revised version of ${{\varvec{y}}}_k$ and adding it into the cluster. Similarly to the previous algorithm, this algorithm is invoked two times, when on the second time is performed on the updated cluster.
  **Intuition and motivation.** This algorithm works similarly to the HR algorithm, however, it adds a few more candidates to the set of candidates. Some of the added candidates may be added more than once. Thus, the algorithm helps assessing the number of time they occur, which is crucial to the selection of the algorithm’s output.
  **Computational Complexity.** Similarly to the VR algorithm, the HR algorithms also works in two iterations of the same form, for any ${{\varvec{y}}}_k \in {{\textbf{C}}}$. Hence, its complexity is ${{\mathcal {O}}}(n^2t^2)$.

The four algorithms described above are invoked by the ITR algorithm on both the original cluster ${{\textbf{C}}}$ and the reversed cluster ${{\textbf{C}}}^R$. Considering also the reversed cluster ${{\textbf{C}}}^R$ can help detect and eliminate the synchronization effect that is created when deletion errors shorten the traces. Thus, the ITR algorithm creates a set of candidates. As mentioned in the beginning of this section, the candidates are evaluated and scored by the ITR algorithm based on their length, number of occurrences and their distance with the original traces in the cluster. Finally, the ITR algorithm returns as an output the candidates with the highest score. This calculation also works in quadratic complexity, and thus the overall complexity of the algorithm is ${{\mathcal {O}}}(n^2t^2)$. The ITR algorithm is applied twice on each cluster. This is because, for the majority of clusters, the set of candidates converges after the second iteration, and further iterations, such as the third, do not lead to improved results.

### The pattern path algorithm (PP Algorithm)

In this section we present the Pattern Path algorithm, or in short the PP Algorithm. The algorithm, described also in Algorithm 1, is the main procedure of the iterative algorithm (ITR Algorithm) that corrects edit errors. Denote by ${{\varvec{w}}}$ an arbitrary LCS sequence of ${{\varvec{x}}}$ and ${{\varvec{y}}}$ of length $\ell$. The sequence ${{\varvec{w}}}$ is a subsequence of ${{\varvec{x}}}$, and hence, all of its $\ell$ symbols appear in some indices of ${{\varvec{x}}}$, and assume these indices are given by $i^{{\varvec{x}}}_1< i^{{\varvec{x}}}_2< \cdots < i^{{\varvec{x}}}_\ell$. It should be noted that a subsequence can have more than one set of such indices, while the number of such sets is defined as the embedding number^42,43^. In our algorithm, we chose one of these sets arbitrarily. Furthermore, given a set of such indices $i^{{\varvec{x}}}_1< i^{{\varvec{x}}}_2< \cdots < i^{{\varvec{x}}}_\ell$, we define the *embedding sequence* of ${{\varvec{w}}}$ in ${{\varvec{x}}}$, denoted by ${{\varvec{u}}}_{{{\varvec{x}}}, {{\varvec{w}}}}$, as a sequence of length $|{{\varvec{x}}}|$ where for $1\leqslant j \leqslant \ell$, ${{\varvec{u}}}_{{{\varvec{x}}}, {{\varvec{w}}}}(i^{{\varvec{x}}}_j)$ equals to ${{\varvec{x}}}(i^{{\varvec{x}}}_j)$ and otherwise it equals to ?.

The *gap* of ${{\varvec{x}}}$, ${{\varvec{y}}}$ and their length-$\ell$ LCS sequence ${{\varvec{w}}}$ in index $1\leqslant j\leqslant |{{\varvec{x}}}|$ with respect to ${{\varvec{u}}}_{{{\varvec{x}}}, {{\varvec{w}}}}$ and ${{\varvec{u}}}_{{{\varvec{y}}}, {{\varvec{w}}}}$, denoted by $\text {gap}_{{{\varvec{u}}}_{{{\varvec{x}}}, {{\varvec{w}}}}}^{{{\varvec{u}}}_{{{\varvec{y}}}, {{\varvec{w}}}}}(j)$, is defined as follows. In case the *j*th or the $(j-1)$th symbol in ${{\varvec{u}}}_{{{\varvec{x}}}, {{\varvec{w}}}}$ equals ?, $\text {gap}_{{{\varvec{u}}}_{{{\varvec{x}}}, {{\varvec{w}}}}}^{{{\varvec{u}}}_{{{\varvec{y}}}, {{\varvec{w}}}}}(j)$ is defined as an empty sequence. Otherwise, the symbol ${{\varvec{u}}}_{{{\varvec{x}}}, {{\varvec{w}}}}(j)$ also appears in ${{\varvec{w}}}$. Denote by $j'$, the index of the symbol ${{\varvec{u}}}_{{{\varvec{x}}}, {{\varvec{w}}}}(j)$ in ${{\varvec{w}}}$. Recall that the sequence ${{\varvec{w}}}$ is an LCS of ${{\varvec{x}}}$ and ${{\varvec{y}}}$, and ${{\varvec{u}}}_{{{\varvec{y}}}, {{\varvec{w}}}}$ is the embedding sequence of ${{\varvec{w}}}$ in ${{\varvec{y}}}$. Given ${{\varvec{u}}}_{{{\varvec{y}}}, {{\varvec{w}}}}$, we can define the sequence of indices $i^{{\varvec{y}}}_1< i^{{\varvec{y}}}_2< \cdots < i^{{\varvec{y}}}_\ell$, such that ${{\varvec{w}}}(j') = {{\varvec{y}}}(i^{{\varvec{y}}}_{j'})$ for $1\leqslant j' \leqslant \ell$. Given such a sequence of indices, $\text {gap}_{{{\varvec{u}}}_{{{\varvec{x}}}, {{\varvec{w}}}}}^{{{\varvec{u}}}_{{{\varvec{y}}}, {{\varvec{w}}}}}(j)$ is defined as the sequence ${{\varvec{y}}}_{[i^{{\varvec{y}}}_{j'-1}+1: i^{{\varvec{y}}}_{j'}-1]}$, which is the sequence between the appearances of the $j'$th and the $(j'-1)$th symbols of ${{\varvec{w}}}$ in ${{\varvec{y}}}$. Note that since $i^{{\varvec{y}}}_{j'}$ can be equal to $i^{{\varvec{y}}}_{j'-1}+1$, $\text {gap}_{{{\varvec{u}}}_{{{\varvec{x}}}, {{\varvec{w}}}}}^{{{\varvec{u}}}_{{{\varvec{y}}}, {{\varvec{w}}}}}(j)$ can be an empty sequence. Roughly speaking, the $\text {gap}_{{{\varvec{u}}}_{{{\varvec{x}}}, {{\varvec{w}}}}}^{{{\varvec{u}}}_{{{\varvec{y}}}, {{\varvec{w}}}}}(j)$ holds every symbol that appears in ${{\varvec{y}}}$ between the $(j'-1)$th and $j'$th symbols of the LCS ${{\varvec{w}}}$, based on the embedding sequence ${{\varvec{u}}}_{{{\varvec{w}}}, {{\varvec{y}}}}$.

The *pattern* of ${{\varvec{x}}}$ and ${{\varvec{y}}}$ with respect to the LCS sequence ${{\varvec{w}}}$, its embedding sequences ${{\varvec{u}}}_{{{\varvec{x}}}, {{\varvec{w}}}}$ and ${{\varvec{u}}}_{{{\varvec{y}}}, {{\varvec{w}}}}$, an index $1\leqslant i \leqslant |{{\varvec{x}}}|$ and a length $m\geqslant 2$, denoted by $Ptn({{\varvec{x}}},{{\varvec{y}}},{{\varvec{w}}},{{\varvec{u}}}_{{{\varvec{x}}}, {{\varvec{w}}}},{{\varvec{u}}}_{{{\varvec{y}}}, {{\varvec{w}}}},i,m)$, is defined as:

$$\begin{aligned} Ptn({{\varvec{x}}},{{\varvec{y}}},{{\varvec{w}}},{{\varvec{u}}}_{{{\varvec{x}}}, {{\varvec{w}}}},{{\varvec{u}}}_{{{\varvec{y}}}, {{\varvec{w}}}},i,m)\hspace{-0.25ex}\triangleq \hspace{-0.25ex} ({{\varvec{u}}}_{{{\varvec{x}}},{{\varvec{w}}}}({i\hspace{-0.25ex}-\hspace{-0.25ex}1}), \text {gap}_{{{\varvec{u}}}_{{{\varvec{x}}}, {{\varvec{w}}}}}^{{{\varvec{u}}}_{{{\varvec{y}}}, {{\varvec{w}}}}}(i), {{\varvec{u}}}_{{{\varvec{x}}},{{\varvec{w}}}}({i}), \ldots , \text {gap}_{{{\varvec{u}}}_{{{\varvec{x}}}, {{\varvec{w}}}}}^{{{\varvec{u}}}_{{{\varvec{y}}}, {{\varvec{w}}}}}(i+m\hspace{-0.25ex}-\hspace{-0.25ex}1), {{\varvec{u}}}_{{{\varvec{x}}},{{\varvec{w}}}}({i\hspace{-0.25ex}+\hspace{-0.25ex}m\hspace{-0.25ex}-\hspace{-0.25ex}1})), \end{aligned}$$

where for $i<1$ and $i>|{{\varvec{x}}}|$, the symbol ${{\varvec{u}}}_{{{\varvec{x}}}, {{\varvec{w}}}}(i)$ is defined as the null character and $\text {gap}_{{{\varvec{u}}}_{{{\varvec{x}}}, {{\varvec{w}}}}}^{{{\varvec{u}}}_{{{\varvec{y}}}, {{\varvec{w}}}}}(i)$ is defined as an empty sequence. The parameter *m* defines the length of the pattern, that is the number of embedding sequences and gaps that comprises the patterns. In our implementation of the algorithm, the length of the patterns is defined as $m=2$.

We also define the prefix and suffix of a pattern $Ptn({{\varvec{x}}},{{\varvec{y}}},{{\varvec{w}}},{{\varvec{u}}}_{{{\varvec{x}}}, {{\varvec{w}}}},{{\varvec{u}}}_{{{\varvec{y}}}, {{\varvec{w}}}},i,m)$ to be:

$$\begin{aligned}{} & {} \text {Prefix}(Ptn({{\varvec{x}}},{{\varvec{y}}},{{\varvec{w}}},{{\varvec{u}}}_{{{\varvec{x}}}, {{\varvec{w}}}},{{\varvec{u}}}_{{{\varvec{y}}}, {{\varvec{w}}}},i,m)) \hspace{-0.25ex}\triangleq \hspace{-0.25ex}({{\varvec{u}}}_{{{\varvec{x}}},{{\varvec{w}}}}({i\hspace{-0.25ex}-\hspace{-0.25ex}1}), \text {gap}_{{{\varvec{u}}}_{{{\varvec{x}}}, {{\varvec{w}}}}}^{{{\varvec{u}}}_{{{\varvec{y}}}, {{\varvec{w}}}}}(i), {{\varvec{u}}}_{{{\varvec{x}}},{{\varvec{w}}}}({i}), \ldots , {{\varvec{u}}}_{{{\varvec{x}}},{{\varvec{w}}}}({i\hspace{-0.25ex}+\hspace{-0.25ex}m\hspace{-0.25ex}-\hspace{-0.25ex}2})),\\{} & {} \text {Suffix}(Ptn({{\varvec{x}}},{{\varvec{y}}},{{\varvec{w}}},{{\varvec{u}}}_{{{\varvec{x}}}, {{\varvec{w}}}},{{\varvec{u}}}_{{{\varvec{y}}}, {{\varvec{w}}}},i,m)\triangleq ({{\varvec{u}}}_{{{\varvec{x}}},{{\varvec{w}}}}({i}), \text {gap}_{{{\varvec{u}}}_{{{\varvec{x}}}, {{\varvec{w}}}}}^{{{\varvec{u}}}_{{{\varvec{y}}}, {{\varvec{w}}}}}(i+1), \ldots , {{\varvec{u}}}_{{{\varvec{x}}},{{\varvec{w}}}}({i+m-1})). \end{aligned}$$

Finally, we define

$$\begin{aligned} P({{\varvec{x}}}, {{\varvec{y}}}, {{\varvec{w}}},{{\varvec{u}}}_{{{\varvec{x}}}, {{\varvec{w}}}}, {{\varvec{u}}}_{{{\varvec{y}}}, {{\varvec{w}}}}, m) \triangleq \{ Ptn({{\varvec{x}}},{{\varvec{y}}},{{\varvec{w}}},{{\varvec{u}}}_{{{\varvec{x}}}, {{\varvec{w}}}},{{\varvec{u}}}_{{{\varvec{y}}}, {{\varvec{w}}}},,i,m): 1\leqslant i \leqslant |{{\varvec{x}}}|\}. \end{aligned}$$

The Pattern Path Algorithm receives a cluster ${{\textbf{C}}}$ of *t* traces and one of the traces in the cluster ${{\varvec{y}}}_k$. First, the algorithm initializes $L[{{\varvec{y}}}_k]$, which is a set of $|{{\varvec{y}}}_k|$ empty lists. For $1 \leqslant i \leqslant |{{\varvec{y}}}_k|$, the *i*th list of $L[{{\varvec{y}}}_k]$ is denoted by $L[{{\varvec{y}}}_k]_i$. The algorithm pairs ${{\varvec{y}}}_k$ with each of the other traces in ${{\textbf{C}}}$. For each pair of traces, ${{\varvec{y}}}_k$ and ${{\varvec{y}}}_h$, the algorithm computes an arbitrary LCS sequence ${{\varvec{w}}}$, and an arbitrary embedding sequence ${{\varvec{u}}}_{{{\varvec{y}}}_k,{{\varvec{w}}}}$. Then it uses ${{\varvec{w}}}$ and ${{\varvec{u}}}_{{{\varvec{y}}}_k,{{\varvec{w}}}}$ to compute $P({{\varvec{y}}}_k, {{\varvec{y}}}_h, {{\varvec{w}}},{{\varvec{u}}}_{{{\varvec{y}}}_k,{{\varvec{w}}}} ,{{\varvec{u}}}_{{{\varvec{y}}}_h,{{\varvec{w}}}}, m)$. For $1 \leqslant i \leqslant |{{\varvec{y}}}_k|$, the algorithm saves $Ptn({{\varvec{y}}}_k, {{\varvec{y}}}_h, {{\varvec{w}}},{{\varvec{u}}}_{{{\varvec{y}}}_k,{{\varvec{w}}}} ,{{\varvec{u}}}_{{{\varvec{y}}}_h,{{\varvec{w}}}},i,m)$ in $L[{{\varvec{y}}}_k]_i$. Then, the algorithm builds the *pattern graph* $G_{pat}({{\varvec{y}}}_k)=(V({{\varvec{y}}}_k),E({{\varvec{y}}}_k))$, which is a directed acyclic graph, and is defined as follows.

1. $$V({{\varvec{y}}}_k)=\{((Ptn({{\varvec{y}}}_k,{{\varvec{y}}}_h,{{\varvec{w}}},{{\varvec{u}}}_{{{\varvec{y}}}_k, {{\varvec{w}}}},{{\varvec{u}}}_{{{\varvec{y}}}_h, {{\varvec{w}}}},i,m), i): 1\leqslant h \leqslant t, h\ne k, 1\leqslant i \leqslant |{{\varvec{y}}}_k| \}\cup \{ S, U\}$$.
  The vertices are pairs of patterns and their index. Note that the same pattern can appear in several vertices with different indices *i*. The value |*V*| equals to the number of distinct pattern-index pairs.
2. $$E ({{\varvec{y}}}_k)\hspace{-.7ex}=\hspace{-.7ex} \{ e\hspace{-.7ex}=\hspace{-.7ex}(v,u)\hspace{-.7ex}:\hspace{-.7ex} v\hspace{-.7ex}=\hspace{-.7ex}(Ptn({{\varvec{y}}}_k,{{\varvec{y}}}_h,{{\varvec{w}}},{{\varvec{u}}}_{{{\varvec{y}}}_k, {{\varvec{w}}}},{{\varvec{u}}}_{{{\varvec{y}}}_h, {{\varvec{w}}}},i,m), i) , u\hspace{-.7ex}=\hspace{-.7ex}(Ptn({{\varvec{y}}}_k,{{\varvec{y}}}_h,{{\varvec{w}}},{{\varvec{u}}}_{{{\varvec{y}}}_k, {{\varvec{w}}}},{{\varvec{u}}}_{{{\varvec{y}}}_h, {{\varvec{w}}}},i\hspace{-.7ex}+\hspace{-.7ex}1,m), i\hspace{-.7ex}+\hspace{-.7ex}1),$$
                      $$\text {Suffix}(Ptn({{\varvec{y}}}_k,{{\varvec{y}}}_h,{{\varvec{w}}},{{\varvec{u}}}_{{{\varvec{y}}}_k, {{\varvec{w}}}},{{\varvec{u}}}_{{{\varvec{y}}}_h, {{\varvec{w}}}},i,m)=\text {Preffix}(Ptn({{\varvec{y}}}_k,{{\varvec{y}}}_h,{{\varvec{w}}},{{\varvec{u}}}_{{{\varvec{y}}}_k, {{\varvec{w}}}},{{\varvec{u}}}_{{{\varvec{y}}}_h, {{\varvec{w}}}},i\hspace{-.7ex}+\hspace{-.7ex}1,m) \}.$$
3. The weights of the edges are defined by $w:E\rightarrow N$ as follows:
  For $e=(v,u)$, where $u=(Ptn({{\varvec{y}}}_k,{{\varvec{y}}}_h,{{\varvec{w}}},{{\varvec{u}}}_{{{\varvec{y}}}_k, {{\varvec{w}}}},{{\varvec{u}}}_{{{\varvec{y}}}_h, {{\varvec{w}}}},i,m),i)$, it holds that
  $$\begin{aligned} w(e)= |\{ Ptn\in L[{{\varvec{y}}}_k]_i: Ptn= Ptn({{\varvec{y}}}_k,{{\varvec{y}}}_h,{{\varvec{w}}},{{\varvec{u}}}_{{{\varvec{y}}}_k, {{\varvec{w}}}},{{\varvec{u}}}_{{{\varvec{y}}}_h, {{\varvec{w}}}},i,m) \} |, \end{aligned}$$
  which is the number of appearances of $Ptn({{\varvec{y}}}_k,{{\varvec{y}}}_h,{{\varvec{w}}},{{\varvec{u}}}_{{{\varvec{y}}}_k, {{\varvec{w}}}},{{\varvec{u}}}_{{{\varvec{y}}}_h, {{\varvec{w}}}},i,m)$ in $L[{{\varvec{y}}}_k]_i$.
4. The vertex *S*, which does not correspond to any pattern, is connected to all vertices of the first index. The weight of these edges is the number of appearances of the incoming vertex pattern.
5. The vertex *U* has incoming edges from all vertices of the last index and the weight of each edge is zero.

Finally, the Pattern Path Algorithm identifies a longest path from *S* to *U* in the graph. This path induces a sequence, denoted by $\widehat{{{\varvec{y}}}}_k$, which is formed by concatenating the patterns of ${{\varvec{y}}}_k$ (including their gaps if such exist), that appears in the vertices of the longest path in the pattern graph. It’s important to note that $\widehat{{{\varvec{y}}}}_k$ represents a modified version of ${{\varvec{y}}}_k$, as it incorporates the patterns present in ${{\varvec{y}}}_k$. The algorithm returns $\widehat{{{\varvec{y}}}}_k$, which is an updated rendition of ${{\varvec{y}}}_k$, while also incorporating any gaps inherited from the vertices along the longest path. To illustrate the Pattern Path Algorithm’s workflow, we provide an example in the following section.

**Algorithm 1:**
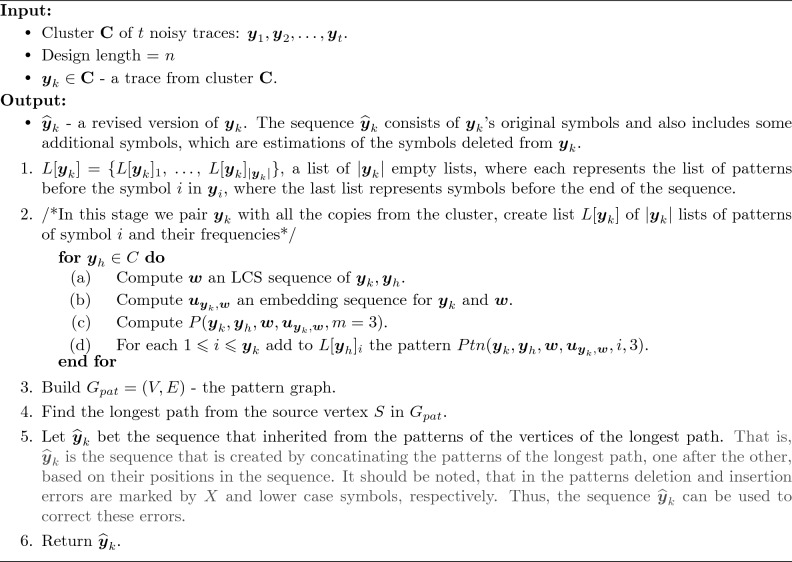
The Pattern Path Algorithm (PP Algorithm)

#### Example 1

***Example of the PP Algorithm***

We present here a short example of the Pattern Path Algorithm and its related definitions.

***Step 1 - Input of the algorithm.*** The original strand in this example is ${{\varvec{x}}}$ which is given below. The cluster of its $t=5$ traces is ${{\textbf{C}}}=\{ {{\varvec{y}}}_1, \ldots , {{\varvec{y}}}_5 \}$. The original design length of ${{\varvec{x}}}$ is $n=10$. The traces are noisy copies of ${{\varvec{x}}}$ and include deletions, insertions, and substitutions. In this example Algorithm 1 receives the cluster ${{\textbf{C}}}$ and the trace ${{\varvec{y}}}_k={{\varvec{y}}}_1$ as its input.

- $${{\varvec{x}}}=$$ GTAGTGCCTG.
- $${{\varvec{y}}}_1=$$ GTAGGTGCCG.
- $${{\varvec{y}}}_2=$$ GTAGTCCTG.
- $${{\varvec{y}}}_3=$$ GTAGTGCCTG.
- $${{\varvec{y}}}_4=$$ GTAGCGCCAG.
- $${{\varvec{y}}}_5=$$ GCATGCTCTG.

**Figure 1:**
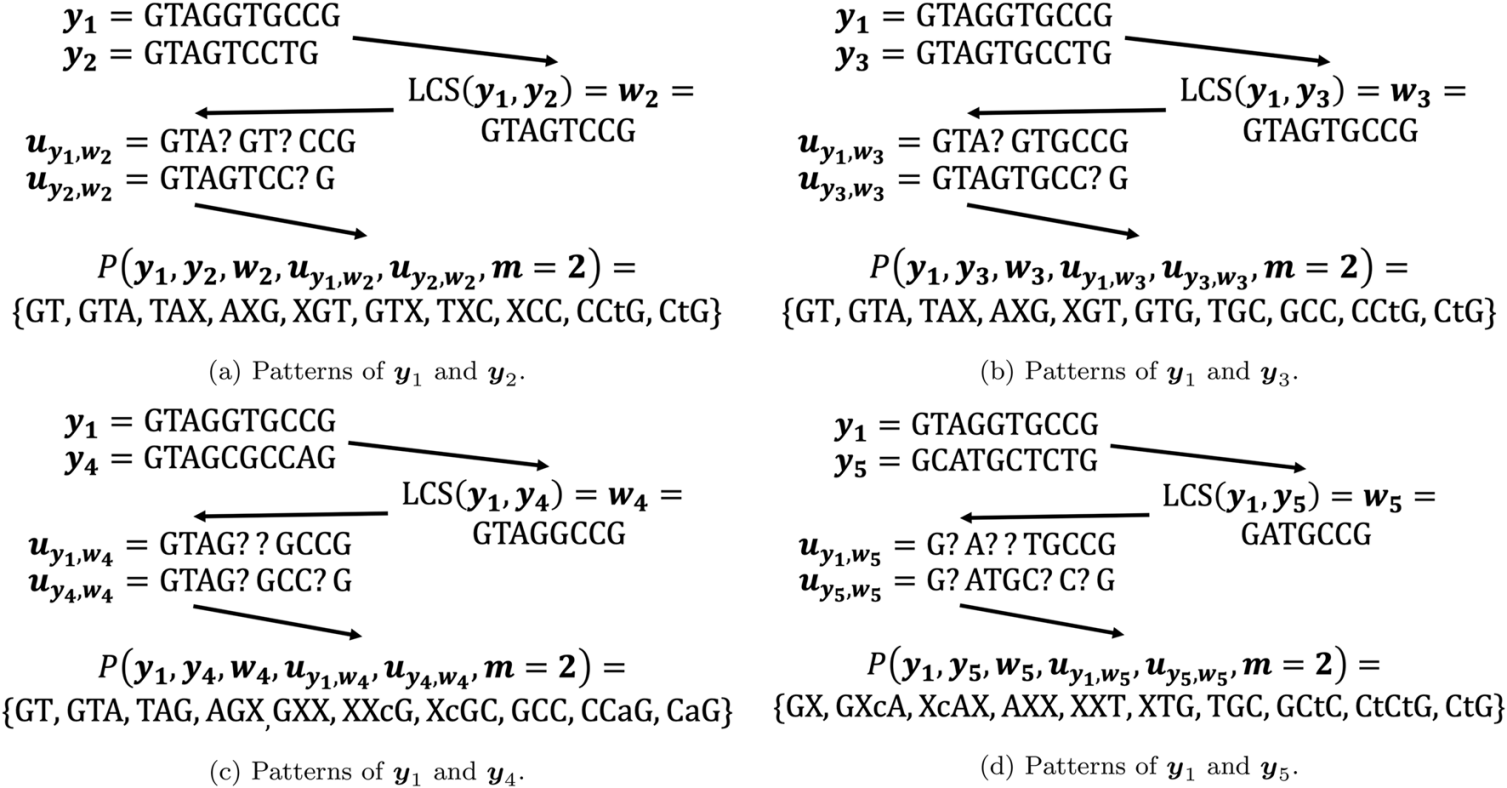
Algorithm 1 Example - Patterns of ${{\varvec{y}}}_1$.

***Step 2 - Computation of the LCSs and the patterns in cluster.*** After receiving the input the PP algorithm continues with the next step of computing the LCSs and the patterns of the cluster. Figure 1 presents the process of computing the patterns of $({{\varvec{y}}}_1, {{\varvec{y}}}_2), ({{\varvec{y}}}_1, {{\varvec{y}}}_3),$ $({{\varvec{y}}}_1, {{\varvec{y}}}_4), ({{\varvec{y}}}_1, {{\varvec{y}}}_5)$. For each pair, ${{\varvec{y}}}_1$ and ${{\varvec{y}}}_i$, Fig. 1 depicts ${{\varvec{w}}}_i$, which is an LCS of the sequences ${{\varvec{y}}}_1$ and ${{\varvec{y}}}_i$. Then, the figure presents ${{\varvec{u}}}_{{{\varvec{y}}}_1, {{\varvec{w}}}_i}$ and ${{\varvec{u}}}_{{{\varvec{y}}}_i, {{\varvec{w}}}_i}$, which are the embedding sequences that the Pattern Path Algorithm uses in order to compute the patterns. Lastly, the list of patterns of each pair is depicted in an increasing order of their indices. Note that lowercase symbols present gaps and *X* presents the symbol ?.

***Step 3 - Evaluating the patterns and their accuracy.*** The following list summarizes the patterns and their frequencies. Each list includes patterns from specific index. The numbers on the right side of each pattern in a list represents the pattern’s frequency.

- $$L[{{\varvec{y}}}_1]_1=\{\text {GT}: 3, \text {GX}: 1\}$$.
- $$L[{{\varvec{y}}}_1]_2=\{\text {GTA}: 3, \text {GXcA}: 1\}$$.
- $$L[{{\varvec{y}}}_1]_3=\{\text {TAX}: 2, \text {TAG}: 1, \text {XcAX}: 1\}$$.
- $$L[{{\varvec{y}}}_1]_4=\{ \text {AXG}: 2, \text {AGX}: 1, \text {AXX}: 1\}$$.
- $$L[{{\varvec{y}}}_1]_5=\{ \text {XGT}: 2, \text {GXX}: 1, \text {XXT}: 1\}$$.
- $$L[{{\varvec{y}}}_1]_6=\{ \text {GTG}:1, \text {GTX}:1, \text {XXcG}:1, \text {XTG}:1\}$$.
- $$L[{{\varvec{y}}}_1]_7=\{ \text {TGC}: 2, \text {TXC}:1, \text {XcGC}: 1\}$$.
- $$L[{{\varvec{y}}}_1]_8=\{ \text {GCC}: 2, \text {XCC}:1, \text {GCtG}:1\}$$.
- $$L[{{\varvec{y}}}_1]_9=\{ \text {CCtG}:2, \text {CCaG}:1. \text {CtCtG}:1\}$$.
- $$L[{{\varvec{y}}}_1]_{10}=\{ \text {CtG}:3, \text {CaG}:1 \}$$.

**Figure 2:**
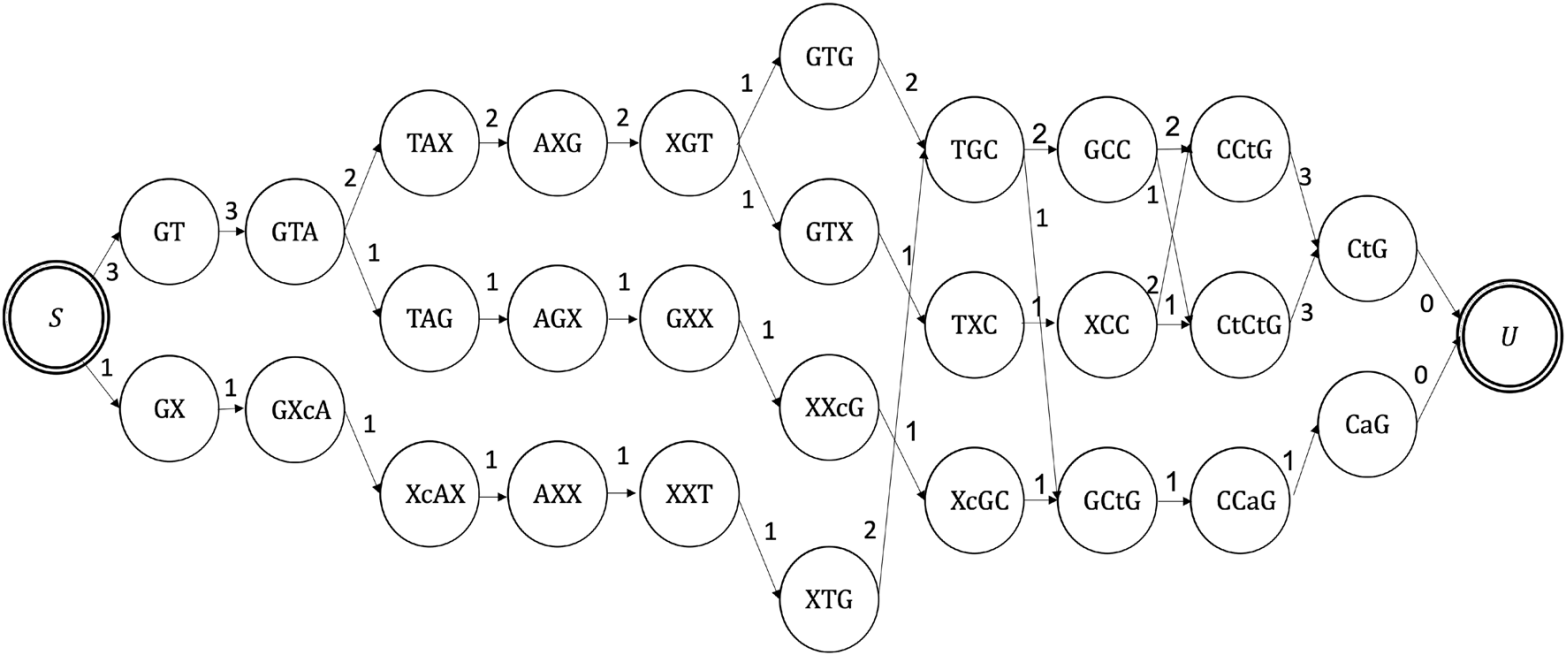
The pattern-path graph.

***Step 4 - Creating the pattern path graph.*** Next, based on the list of patterns above, the pattern path is created. it can be shown that every pattern is a vertex in the graph, and that is represented by its sequence and its position in the sequence ($1\leqslant i \leqslant 10)$. Furthermore, the weights of the edges in the graph represents the frequencies of the patterns. As can be seen in Fig. 2, the created graph is a directed acyclic graph.

***Step 5 - Output.***

It is not hard to observe that the longest path in the pattern path graph of this example is:

$$\begin{aligned} S \hspace{-.75ex} \rightarrow \hspace{-.75ex} GT \hspace{-.75ex} \rightarrow \hspace{-.75ex} GTA \hspace{-.75ex} \rightarrow \hspace{-.75ex} TAX \hspace{-.75ex} \rightarrow \hspace{-.75ex} AXG \hspace{-.75ex} \rightarrow \hspace{-.75ex} XGT \hspace{-.75ex} \rightarrow \hspace{-.75ex} GTG \rightarrow \hspace{-.75ex} TGC \hspace{-.75ex} \rightarrow \hspace{-.75ex} GCC \hspace{-.75ex} \rightarrow \hspace{-.75ex} CCtG \hspace{-.75ex} \rightarrow \hspace{-.75ex} CtG \hspace{-.75ex} \rightarrow \hspace{-.75ex} U, \end{aligned}$$

and the algorithm output will be $\widehat{{{\varvec{y}}}}_1=GTAGTGCCTG={{\varvec{x}}}$.

### The divider BMA algorithm

In this section we shortly describe the *Divider BMA algorithm*, our own variation to the BMA-Lookahead algorithm presented in^33^, that can improve results on large clusters. This algorithm is a stand-alone algorithm, designed for cases in which the clusters are relatively large and have lower error rates with shorter run time on such clusters (compared to the ITR algorithm and the PP algorithm). The Divider BMA algorithm receives a cluster and the design length *n*. The Divider BMA algorithm divides the traces of the cluster into three sub-clusters by their lengths, traces of length *n*, traces of length smaller than *n*, and traces of length larger than *n*. It performs a majority vote on the traces of length *n*. Then, similarly to the technique presented in the BMA algorithm^12^ and in^33^, the Divider BMA algorithm performs a majority vote on the sub-cluster of traces of length smaller than *n*, while detecting and correcting deletion errors. Lastly, the Divider BMA algorithm uses the same technique on the traces of length larger than *n* to detect and correct insertion errors. The time complexity of this algorithm is linear.

## Results

In this section we present an evaluation of the accuracy of our algorithms on our new data set and on data from previous DNA storage experiments^18–20,28,39^. The presented algorithms include the ITR algorithm, which uses the Pattern Path Algorithm (Algorithm 1) along with other algorithms that are described in the supplmentary material. We also implemented the linear-time algorithm from^33^, in the implementation we used several different value of the window size parameter of the algorithm, denoted by *w*. That is, our results include the algorithm from^33^ with $2\leqslant w \leqslant 4$. Additionally, we also implemented the algorithm from^32^, with parameters of $\ell =5$, $\delta =(1+p_s)/2$, $r=2$ and $\gamma =3/4$, while for the data of the DNA storage experiments the substitution probability $p_s$ was taken from^29^. For further information about the parameters we used in the algorithms from^32,33^, the reader is referred to their original papers. Finally, we also included an additional algorithm which is our variation of the BMA algorithm^12,33^ to support also insertion and substitution errors, which is referred by the Divider BMA Algorithm.

In our new dataset, we designed 1000 random strands, each of length 128 symbols, and we matched each one of the strands with an index of length 12. The synthesis of this dataset was done by Twist Bioscience. The synthesized strands were amplified using 8 PCR cycles with the enzyme NebNext *Q*5 high-fidelity DNA polymerase. The sequencing was done using Oxford MinION, with the LSK109 ligation kit. The clustering of the data was done with the SOLQC tool^29^ by binning the reads by their indices. The error characterization was also done using the SOLQC tool^29^. The characterization process was done by calculating the edit distance of the traces and their original design sequence (which is given to SOLQC as input for error characterization). For further details about the clustering process and the error characterization process please see^29^. The inspected error rate of the data is $3.5\%$. Additionally, we also evaluated our algorithm on a new data set that is based on the design from^39^. We re-ordered the same design sequences as in^39^ from Twist Bioscience. Then we amplified the synthesized DNA molecules with 8 PCR cycles using the *Q*5 enzyme and performed the sequencing using Oxford MinION, with the LSK110 ligation kit. According to our analysis the error rate in this data set is $3.41\%$.

**Figure 3:**
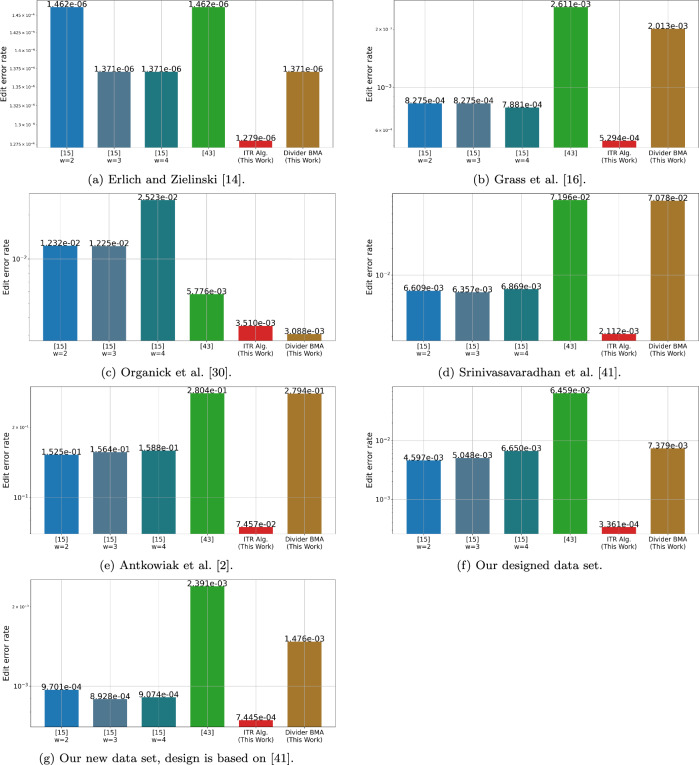
Edit error rate by the reconstruction algorithm, the results are presented on our new designed data sets and on data from DNA storage experiments^18–20,28,39^.

Figure 3 presents the results of the tested algorithms on data from previous DNA storage experiments^18–20,28,39^ as well as on our new designed dataset. The clustering of these data sets was made by the SOLQC tool^29^. We performed each of the tested algorithms on the data and evaluated the edit error rates. Note that in order to reduce the runtime of the ITR algorithm we filtered clusters of size $t>25$ to have only the first 25 traces. The ITR algorithm presented the lowest edit error rates in almost all of the tested data sets.

We also evaluated the accuracy of our algorithms by simulations. First, we present our interpretation of the insertion-deletion-substitution channel, in which the sequence is transmitted symbol-by-symbol. First, before transmitting the symbol, it checks for an insertion error before the transmitted symbol. The channel flips a coin, and with probability $p_i$, an insertion error occurs before the transmitted symbol. If an insertion error occurs, the inserted symbol is chosen uniformly. Then, the channel checks for a deletion error, and again flips a coin, and with probability $p_d$ the transmitted symbol is deleted. Lastly, the channel checks for a substitution error. The channel flips a coin, and with probability $p_s$ the transmitted symbol is substituted to another symbol. The substituted symbol is chosen uniformly. In case that both deletion and substitution errors occurs in the same symbol, we refer to it as a substitution.

**Figure 4:**
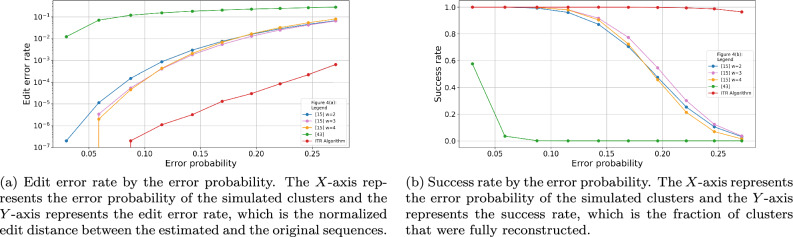
Edit error rate and success rate by the error probabilities for cluster size of $t=20$. The length of the original sequence was $n=100$ and the error probabilities ranges between 0.029701 and 0.271.

We simulated 100,000 clusters of size $t=10,20$, the sequences length was $n=100$, and the alphabet size was $q=4$. The deletion, insertion, and substitution probabilities were all identical, and ranged between 0.01 and 0.1. It means that the actual error probability of each symbol was $1-(1-p_i)(1-p_s)(1-p_d)$ and ranged between 0.029701 and 0.271. We reconstructed the original sequences of the clusters using the ITR Algorithm and the algorithms from^33^ and from^32^. For each algorithm we evaluated its edit error rate and the success rate which is the fraction of clusters that were reconstructed successfully with no error. The edit error rate of the ITR Algorithm was the lowest among the tested algorithms, while the algorithm from^32^ presented the highest edit error rates. Moreover, it can be seen that the ITR Algorithm presented significantly low edit error rates for higher values of error probabilities. Such high probabilities can reflect the error probabilities of light-directed array-based synthesis as reported in^28^. The results on the simulated data for $t=20$ are depicted in Fig. 4.

Additionally, we performed another simulation to study the accuracy of our algorithms on the expected near-future DNA synthesis technologies. To do so, we simulated 10, 000 clusters of sequences of length $n=300$. For each cluster, we simulated $t=20$ noisy traces as described in the previous paragraph. Finally, we evaluate the accuracy of our algorithm on these data sets. In all cases the ITR algorithm shows significantly lower error rates compare to any of the other tested algorithms. The results are summarized in Fig. 6.

**Figure 5:**
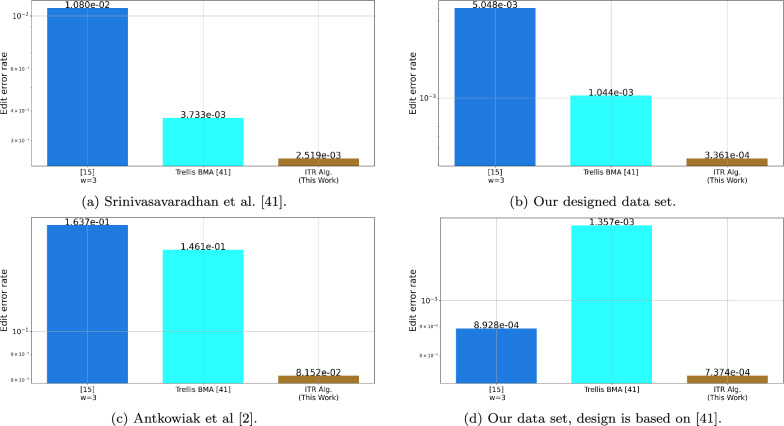
Edit error rate by the reconstruction algorithm on small clusters, the results are presented on our new designed data set and on data from DNA storage experiments^28,39^.

Lastly, we compared our algorithms with the Trellis BMA algorithm that was published in^39^. This algorithm was designed to support data sets with higher error rate and hence we evaluated it on the data sets that presented relatively higher rates. These data sets include our new designed data sets, and the data set from^28,39^. Similarly to what the authors suggested in^39^, to improve the accuracy and the running time of the algorithm and to ensure fair comparison of this algorithm, we evaluated it on clusters of size 10 or less. Thus, we randomly sampled 10 traces from each of the clusters in these data sets and then invoked the algorithms on them. For clusters of size 10 or less we simply ran the algorithms on the entire cluster. The error rates of the algorithms are described in Fig. 5, where it can be seen that the ITR algorithm shows less error rates compared to the Trellis BMA algorithm^39^ and the BMA Lookahead algorithm^33^.

**Figure 6:**
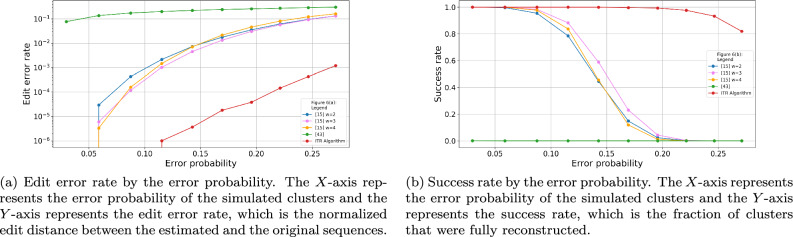
Edit error rate and success rate by the error probabilities for cluster size of $t=20$. The length of the original sequence was $n=300$ and the error probabilities ranges between 0.029701 and 0.271.

### Performance evaluation

We evaluate the performance of the different algorithms discussed in this paper. The performance evaluation was performed on our server with Intel(R) Xeon(R) CPU E5-2630 v3 2.40 GHz. To test their performances we implemented our algorithms as well as the previously published algorithm from^33^ and from^39^, which presented the second-lowest error rates in our results from “Results” section. In order to present reliable performance evaluation, the clusters in our experiments were reconstructed in serial order. However, it is important to note, that for practical uses, additional performance improvements can be made by performing the algorithms on the different clusters in parallel and shortening the running time.

- Experiment A evaluates the running time of the algorithms on simulated data of 450 clusters of sequence length of $n=110$, $p_d=p_s=p_i=0.05$, so the total error rate was 0.142625. The cluster sizes were distributed normally between $t=1$ and $t=30$. The algorithm from^33^ (with parameter $w=3$) reconstructed the full data set in 0.6 second with total error rate of 0.02111, and success rate of 0.531. The ITR algorithm reconstructed the 450 clusters in 683 seconds and presented significantly less errors with total edit error rate of 0.000566, and success rate of 0.982262.
- Experiment B studies the running time of the reconstruction of the data set from^39^. This data set includes 9984 clusters. Algorithm achieved a success rate of 0.875714, error rate of 0.00211 with total run time of 11, 486 seconds, and reported error rate of roughly $7\%$. The algorithm from^33^ (with parameter $w=3$) was able to reconstruct this data set in 12 seconds, while achieving three times larger error rate of 0.006 and success rate of 0.779948. It should be further noted that the runtime of the Trellis BMA algorirthm, that was presented in^39^ is significantly larger when running serially, and takes more than 10 days.
- Experiment C consists of the first 100,000 clusters from the data set of^20^, and measures the running time of their reconstruction. The ITR algorithm was able to achieve a success rate of 0.99788 and an error rate of 0.0000282 in 93, 927 seconds, while the algorithm from^33^ (with parameter $w=3$) reconstructed the clusters in 79 seconds, while presenting success rate of 0.9979 and total edit error rate of 0.000047, which is approximately 1.6 times more errors compared to the ITR algorithm. Since for this data set, the divider BMA presented lower error rate compare to^33^, we decided to evaluate its performance on this data set. The divider BMA algorithm reconstructed the data set in 64 seconds and error rate of 0.000044.

It can be seen that, the run times of the ITR algorithm is remarkably longer. However, they can significantly improve both the success rate and the error rate of the algorithm from^33^. Their advantage is even larger for small clusters as in experiment A, and when the error rates are relatively high as in experiments A and in^39^. Hence, it is also possible to consider hybrid algorithm that invokes the ITR algorithm or the the algorithm^33^ based on the given cluster sizes. Since in data from previous DNA storage experiments the variance in the cluster size and in the error rates can be really high^29^, we added an additional condition to the hybrid algorithm, so it invokes the algorithm from^33^ if the cluster is of size 20 or larger, or if the absolute distance of the difference between the average length of the traces in the cluster and the design length is larger than 10% of the design length. The hybrid algorithm reconstructed the first 100,000 clusters of^20^ (experiment C) in 58 seconds and presented error rate of 0.000333 are the largest success rate among all tested algorithm of 0.998. The results of the performance experiments are also depicted in Table 1.

**Table 1:** Performance evaluation of the ITR Algorithm, Gopalan et al. algorithm^33^, and the Hybrid Algorithm. The number presented the running time in seconds and the error rates of each of the algorithms for each of the experiments.

	ITR algorithm	Gopalan et al.^33^	Hybrid algorithm
Experiment A - time (sec.)	683	0.6	155
Experiment A - error rate	0.000566	0.02111	0.0121414
Experiment A - success rate	0.982	0.531	0.695
Experiment B^39^- time (sec.)	11,486	12	401
Experiment B^39^- error rate	0.00211	0.006	0.005
Experiment B^39^- success rate	0.875714	0.779948	0.8002
Experiment C^20^- time (sec.)	93,927	78	58
Experiment C^20^- error rate	0.0000282	0.000047	0.000033
Experiment C^20^- success rate	0.9979	0.9979	0.998

We also study the running time of our algorithms on simulated data designed to resemble the near-future synthesis technologies in Experiment D. In this test, we assess 10,000 clusters, generated from random DNA sequences of length $n=300$. In this scenario, the simulated error probabilities are set to be $p_d=p_s=p_i=0.05$. Since we also wanted to compare our results on these clusters with the results of the Trellis BMA algorithm^39^, we had to restrict the cluster size to be $t=10$ (to ensure acceptable running time), meaning each cluster in this simulation includes 10 traces. Given this cluster size, the hybrid algorithm cannot significantly enhance the running time of the ITR algorithm. Therefore, in this evaluation, we compare the ITR algorithm, the Trellis BMA algorithm^39^, and the BMA Lookahead algorithm^33^. We observe that the ITR shows the higher success rate and the lower error rate compared to the tested algorithms. Additionally, the second best algorithm in terms of accuracy is the Trellis BMA^39^ that shows running time which is more than ten times higher compared to the ITR algorithm. It should be noted that during the listed running time of 864,000 seconds, the Trellis BMA^39^ was not able to reconstruct the entire set of 10,000 clusters, but only a fraction of the first 1,000 clusters. Thus, the actual running time of this algorithm is much higher than listed in the table. The success rate of the BMA Lookahead algorithm^33^ is the lowest among the tested algorithm, and thus we believe it might not be applicable to the near-future technologies. The results of this experiment are summarized in Table 2.

**Table 2:** Performance evaluation of the ITR Algorithm, Gopalan et al. algorithm^33^, and the Trellis BMA Algorithm^39^. The number presented the running time in seconds and the error rates of each of the algorithms for each of the experiments.

	ITR Algorithm	Gopalan et al.^33^	Trellis BMA^39^
Experiment D - time (sec.)	80,301	59.7	864,000
Experiment D - error rate	0.00057	0.0324	0.0044
Experiment D - success rate	0.8955	0.037	0.5496

## Conclusions and discussion

We presented in this paper several new algorithms for the DNA reconstruction problem. While most of the previously published algorithms either use a symbol-wise majority approaches or have a prior-knowledge of the error rates, our algorithms look globally on the entire sequence of the traces, and use the LCS or SCS of a given set of traces. Our algorithms are designed to specifically support DNA storage systems and to reduce the edit error rate of the reconstructed sequences. According to our tests on simulated data and on data from DNA storage experiments, we found out that our algorithms significantly improved the accuracy compared to the previously published algorithms. While the ITR algorithm’s running time is longer than that of previously published algorithms^12,33^, it still stands as a significantly more efficient choice than the Trellis BMA algorithm^39^. Furthermore, the ITR algorithm demonstrates substantial accuracy improvements, especially in datasets featuring high error rates, smaller clusters, and longer sequences. Nevertheless, for the broader application of DNA storage systems, future research should focus on reducing latency in synthesis, sequencing, clustering, and reconstruction processes. Additionally, this future research should also consider optimizing the ITR algorithm’s running time through parallelization of its steps.

### Supplementary Information

Supplementary Information.

## Data Availability

The datasets generated during the current study are available in the google drive repository, as in the following https://drive.google.com/drive/folders/1c3kopMcUsW_tYnjfgjPLuMaLv-cDBV8O?usp=sharing.

## Code availability

Implementation of the algorithms and instructions on how to use them can be found in the GitHub repository in the following https://github.com/omersabary/Reconstruction. For commercial use please contact the authors. 
