# Supplementary material for: Reconstruction algorithms for DNA-storage systems
Source: Sci Rep. 2024 Jan 23;14:1951. doi: 10.1038/s41598-024-51730-3 (PMC10806084; doi:10.1038/s41598-024-51730-3)
Supplement: Supplementary file 1 — Supplementary Information. [file 41598_2024_51730_MOESM1_ESM.pdf]

Reconstruction Algorithms for DNA-Storage Systems Supplementary Information

Omer Sabary[1], Alexander Yucovich[1], Guy Shapira[1], and Eitan Yaakobi[1]

[1]The Henry & Marilyn Taub Faculty of Computer Science, Technion, Haifa, 3200003, Israel.

1 The Deletion DNA Reconstruction Problem

This section studies the deletion DNA reconstruction problem. Assume that a cluster consists of t traces, $\boldsymbol{y}_1, \boldsymbol{y}_2, \ldots, \boldsymbol{y}_t$, where all of them are noisy copies of a synthesized strand. This model assumes that every strand is a sequence that is independently received by the transmission of a length-n sequence $\boldsymbol{x}$ (the synthesized strand) through a deletion channel with some fixed deletion probability p_d. Our goal is to propose an efficient algorithm which returns $\widehat{\boldsymbol{x}}$, an estimation of the transmitted sequence $\boldsymbol{x}$, with the intention of minimizing $d_L(\boldsymbol{x}, \widehat{\boldsymbol{x}})$, which is the Levenshtein distance between $\boldsymbol{x}$ and $\widehat{\boldsymbol{x}}$. We consider both cases when t is a fixed small number and large values of t. Our approach is based on the maximum likelihood decoder over the deletion channel as presented in [8, 9]. Hence, a formal definition of this decoder is given below.

1.1 Maximum Likelihood Decoder for Multiple Deletion Channels

Consider a channel S that is characterized by a conditional probability Pr_S, which is defined by

$$\mathsf{Pr}_\mathsf{S}\{\boldsymbol{y} \text{ rec. } |\boldsymbol{x} \text{ trans.}\},$$

for every pair $(\boldsymbol{x}, \boldsymbol{y}) \in (\Sigma_q^*)^2$. Note that it is not assumed that the lengths of the input and output sequences are the same as we consider also deletions and insertions of symbols. As an example, it is well known that if S is the *binary symmetric channel* (*BSC*) with crossover probability $0 \leqslant p \leqslant 1/2$, denoted by $\mathsf{BSC}(p)$, it holds that $\mathsf{Pr}_{\mathsf{BSC}(p)}\{\boldsymbol{y} \text{ rec. } |\boldsymbol{x} \text{ trans.}\} = p^{d_H(\boldsymbol{y},\boldsymbol{x})}(1-p)^{n-d_H(\boldsymbol{y},\boldsymbol{x})}$, for all $(\boldsymbol{x}, \boldsymbol{y}) \in (\Sigma_2^n)^2$, and otherwise (the lengths of $\boldsymbol{x}$ and $\boldsymbol{y}$ is not the same) this probability equals 0.

The *maximum-likelihood* (*ML*) *decoder* for a code $\mathcal{C}$ with respect to S, denoted by $\mathcal{D}_{\mathsf{ML}}$, outputs a codeword $\boldsymbol{c} \in \mathcal{C}$ that maximizes the probability $\mathsf{Pr}_\mathsf{S}\{\boldsymbol{y} \text{ rec. } |\boldsymbol{c} \text{ trans.}\}$. That is, for $\boldsymbol{y} \in \Sigma_q^*$,

$$\mathcal{D}_{\mathsf{ML}}(\boldsymbol{y}) = \arg\max_{\boldsymbol{c} \in \mathcal{C}} \left\{\mathsf{Pr}_\mathsf{S}\{\boldsymbol{y} \text{ rec. } |\boldsymbol{c} \text{ trans.}\}\right\}.$$

It is well known that for the BSC, the ML decoder simply chooses the closest codeword with respect to the Hamming distance.

The conventional setup of channel transmission is extended to the case of more than a single instance of the channel. Assume a sequence $\boldsymbol{x}$ is transmitted over some t identical channels of S and the decoder receives all channel outputs $\boldsymbol{y}_1, \ldots, \boldsymbol{y}_t$. This setup is characterized by the conditional probability

$$\mathsf{Pr}_{(\mathsf{S},t)}\{\boldsymbol{y}_1, \ldots, \boldsymbol{y}_t \text{ rec.}|\boldsymbol{x} \text{ trans.}\} = \prod_{i=1}^{t} \mathsf{Pr}_\mathsf{S}\{\boldsymbol{y}_i \text{ rec.}|\boldsymbol{x} \text{ trans.}\}.$$

Now, the input to the ML decoder is the sequences $\boldsymbol{y}_1, \ldots, \boldsymbol{y}_t$ and the output is the codeword $\boldsymbol{c}$ which maximizes the probability $\mathsf{Pr}_{(\mathsf{S},t)}\{\boldsymbol{y}_1, \ldots, \boldsymbol{y}_t \text{ rec.}|\boldsymbol{x} \text{ trans.}\}$.

For two sequences $\boldsymbol{x}, \boldsymbol{y} \in \Sigma_q^*$, the number of times that $\boldsymbol{y}$ can be received as a subsequence of $\boldsymbol{x}$ is called the *embedding number of* $\boldsymbol{y}$ *in* $\boldsymbol{x}$ and is defined by

$$\mathsf{Emb}(\boldsymbol{x}; \boldsymbol{y}) = |\{I \subseteq [|\boldsymbol{x}|] \mid \boldsymbol{x}_I = \boldsymbol{y}\}|.$$

Note that if $\boldsymbol{y}$ is not a subsequence of $\boldsymbol{x}$ then $\mathsf{Emb}(\boldsymbol{x};\boldsymbol{y}) = 0$. The embedding number has been studied in several previous works; see e.g. [1, 3] and in [9] it was referred to as the *binomial coefficient*. In particular, this value can be computed with quadratic complexity [3].

While the calculation of the conditional probability $\mathsf{Pr}_{\mathsf{S}}\{\boldsymbol{y} \text{ rec. } |\boldsymbol{x} \text{ trans.}\}$ is a rather simple task for many of the known channels, it is not straightforward for channels which introduce insertions and deletions. In the *deletion channel* with deletion probability p, denoted by $\mathsf{Del}(p)$, every symbol of the word $\boldsymbol{x}$ is deleted with probability p. For the deletion channel it is known, see e.g. [8, 9], that for all $(\boldsymbol{x},\boldsymbol{y}) \in (\Sigma_q^*)^2$, it holds that

$$\mathsf{Pr}_{\mathsf{Del}(p)}\{\boldsymbol{y} \text{ rec. } |\boldsymbol{x} \text{ trans.}\} = p^{|\boldsymbol{x}|-|\boldsymbol{y}|} \cdot (1-p)^{|\boldsymbol{y}|} \cdot \mathsf{Emb}(\boldsymbol{x};\boldsymbol{y}).$$

According to this property, the ML decoder for one or multiple deletion channels is stated as follows [8].

Lemma 1. *Assume $\boldsymbol{c} \in \mathcal{C} \subseteq (\Sigma_q)^n$ is the transmitted sequence and $\boldsymbol{y}_1,\ldots,\boldsymbol{y}_t \in (\Sigma_q)^*$ are the output sequences from* $\mathsf{Del}(p)$*, then*

$$\mathcal{D}_{\mathsf{ML}}(\boldsymbol{y}_1,\ldots,\boldsymbol{y}_t) = \underset{\boldsymbol{x}\in\mathcal{S}\mathsf{CS}(\boldsymbol{y}_1,\ldots,\boldsymbol{y}_t)}{\arg\max} \left\{ \prod_{i=1}^{t} \mathsf{Emb}(\boldsymbol{x};\boldsymbol{y}_i) \cdot (1-p)^{|\boldsymbol{y}_i|} \cdot p^{|\boldsymbol{x}|-|\boldsymbol{y}_i|} \right\}.$$

Note that since there is more than a single channel, when the goal is to minimize the average decoding error probability, the ML decoder does not necessarily have to output a codeword but any sequence that minimizes the average decoding error probability. In the next sections it will be shown how to use the concepts of the SCS and LCS together with the maximum likelihood decoder in order to build decoding algorithms for the deletion DNA reconstruction and the DNA reconstruction problems.

1.2 An Algorithm for Small Fixed Values of t

A straightforward implementation of this approach on a cluster of size t is to compute the set of shortest common supersequences of $\boldsymbol{y}_1,\boldsymbol{y}_2,\ldots,\boldsymbol{y}_t$, i.e., the set $\mathcal{S}\mathsf{CS}(\boldsymbol{y}_1,\boldsymbol{y}_2,\ldots,\boldsymbol{y}_t)$, and then return the maximum likelihood sequence among them. This algorithm has been rigorously studied in [8] to analyze its Levenshtein error rate for $t = 2$. The method to calculate the length of the SCS commonly uses dynamic programming [5] and its complexity is the product of the lengths of all sequences. Hence, even for moderate cluster sizes, e.g. $t \geqslant 5$, this solution will incur high complexity and impractical running times. However, for many practical values of n and p_d, the original sequence $\boldsymbol{x}$ can be found among the list of SCSs while taking less than t traces or even only two of them. This fact, which we verified empirically, can drastically reduce the complexity of the ML-based algorithm. Furthermore, note that $\boldsymbol{x}$ is always a common supersequence of all traces, however it is not necessarily the shortest one. Hence, our algorithm works as follows. The algorithm creates sorted sets of r-tuples, where each tuple consists of r traces from the cluster. The r-tuples are sorted in a non-decreasing order according to the sum of their lengths. For each r-tuple $(\boldsymbol{y}_{i_1},\ldots,\boldsymbol{y}_{i_r})$, the algorithm first calculates its length of the SCS, i.e., the value $\mathsf{SCS}(\boldsymbol{y}_{i_1},\ldots,\boldsymbol{y}_{i_r})$. Observe that if $\mathsf{SCS}(\boldsymbol{y}_{i_1},\ldots,\boldsymbol{y}_{i_r}) = n$ then the sequence $\boldsymbol{x}$ necessarily appears in the set of SCSs of $(\boldsymbol{y}_{i_1},\ldots,\boldsymbol{y}_{i_r})$, that is, $\boldsymbol{x} \in \mathcal{S}\mathsf{CS}(\boldsymbol{y}_{i_1},\ldots,\boldsymbol{y}_{i_r})$. However it is not necessarily the only sequence in $\mathcal{S}\mathsf{CS}(\boldsymbol{y}_{i_1},\ldots,\boldsymbol{y}_{i_r})$. Hence, all is left to do is to filter the set $\mathcal{S}\mathsf{CS}(\boldsymbol{y}_{i_1},\ldots,\boldsymbol{y}_{i_r})$ with sequences that are supersequences of all t traces and finally return the maximum likelihood among them. The algorithm iterates over all possible r-tuples for $r = 2, 3, 4$ and if none of them succeeds, the algorithm computes all SCSs of maximal length that were observed throughout its run and returns the one that minimizes the sum of Levenshtein distances from all copies in the cluster.

In Algorithm 1, we present a pseudo-code of our solution for the deletion DNA reconstruction problem. Note that the algorithm uses another procedure which is presented in Algorithm 2 to filter the supersequences and output the maximum likelihood supersequence. The input to the algorithm is the length n of the original sequence, and a cluster of t traces $\mathbf{C}$. Algorithm 1's main loop is in Step 2; first in Step 2-a it generates the set F, which is a sorted set of all r-tuples of traces by the sum of their lengths. Then, in Step 2-b it iterates over all r-tuples in F and checks for each r-tuple, $(\boldsymbol{y}_{i_1},\ldots,\boldsymbol{y}_{i_r})$, if the length of their SCS, i.e., $\mathsf{SCS}(\boldsymbol{y}_{i_1},\ldots,\boldsymbol{y}_{i_r})$, equals n. If it is equal to n, it computes the set of all its SCSs, $\mathcal{S}\mathsf{CS}(\boldsymbol{y}_{i_1},\ldots,\boldsymbol{y}_{i_r})$, and invokes Algorithm 2. Algorithm 2 checks if one or more of those SCSs are supersequences of all of the traces in the cluster, and if so it returns the maximum likelihood among them. In case that $\mathsf{SCS}(\boldsymbol{y}_{i_1},\ldots,\boldsymbol{y}_{i_r}) < n$, the algorithm checks also if it is equal or greater than $n_{\max}$, which is the longest SCS that was found so far. In this case, the algorithm saves $\mathbf{C}_{\max}$, which is the set of all r-tuples such that the length of their SCS equals $n_{\max}$. In Step 3, the algorithm computes $\mathbf{S}_{\max} = \bigcup_{\boldsymbol{c}\in\mathbf{C}_{\max}} \mathcal{S}\mathsf{CS}(\boldsymbol{c})$, which is the union of sets of SCSs of the r-tuples that the length of their SCS was $n_{\max}$. In Step 4, the algorithm invokes again Algorithm 2 to

check if $\mathbf{S}_{\max}$ includes supersequences of all traces in $\mathbf{C}$ and returns the maximum likelihood among them. If none of the sequences in $\mathbf{S}_{\max}$ is a supersequence of all traces in $\mathbf{C}$, the algorithm returns in Step 5 the sequence which minimizes the sum of Levenshtein distances to all the traces in $\mathbf{C}$.

Algorithm 1 ML-SCS Reconstruction

Input:

- Cluster $\mathbf{C}$ of t noisy traces: $\boldsymbol{y}_1, \boldsymbol{y}_2, \ldots, \boldsymbol{y}_t$ sorted by their lengths from the longest to the shortest.
- Design length= n.

Output: $\widehat{\boldsymbol{x}}$ - Estimation of the original sequence.

1. $\widehat{\boldsymbol{x}} = \epsilon$, $n_{\max} = 0$, $\mathbf{C}_{\max} = \emptyset$.
2. **for** r = 2, 3, 4 **do**
   (a) Denote F=$\{\boldsymbol{c}_i^{(r)} = (\boldsymbol{y}_{i_1}, \boldsymbol{y}_{i_2}, \ldots, \boldsymbol{y}_{i_r}) | 1 \leqslant i \leqslant \binom{t}{r}, 1 \leqslant i_1 < i_2 < \cdots < i_r \leqslant t\}$ the set of all r-tuples from $\mathbf{C}$, sorted by non-decreasing order of the sum of the lengths of the copies in each tuple.

   (b) **for** i = 1, 2, ..., $\binom{t}{r}$ **do**
      if $\mathsf{SCS}(\boldsymbol{c}_i^{(r)}) = n$ **then**
         $\mathbf{S} = \mathcal{S}\mathsf{CS}(\boldsymbol{c}_i^{(r)})$
         $\widehat{\boldsymbol{x}}$ = ML-Supersequence($\mathbf{S}, \mathbf{C}$)
         if $\widehat{\boldsymbol{x}} \neq \epsilon$ **then**
            return $\widehat{\boldsymbol{x}}$
         end if
      else
         if $\mathsf{SCS}(\boldsymbol{c}_i^{(r)}) > n_{\max}$ **then**
            $n_{\max} = \mathsf{SCS}(\boldsymbol{c}_i^{(r)})$
            $\mathbf{C}_{\max} = \{\boldsymbol{c}_i^{(r)}\}$
         end if
         if $\mathsf{SCS}(\boldsymbol{c}_i^{(r)}) = n_{\max}$ **then**
            $\mathbf{C}_{\max} = \mathbf{C}_{\max} \cup \{\boldsymbol{c}_i^{(r)}\}$
         end if
      end if

   (c) **end for**
   end for
3. Compute $\mathbf{S}_{\max} = \bigcup_{\boldsymbol{c} \in \mathbf{C}_{\max}} \mathcal{S}\mathsf{CS}(\boldsymbol{c})$, the union of all $\mathcal{S}\mathsf{CS}$ of $\boldsymbol{c}_i^{(r)} \in \mathbf{C}_{\max}$.
4. $\widehat{\boldsymbol{x}}$ = ML-Supersequence($\mathbf{S}_{\max}, \mathbf{C}$)
5. **if** $\widehat{\boldsymbol{x}} \neq \epsilon$ **then**
      return $\widehat{\boldsymbol{x}}$
   else
      Return the sequence from $\mathbf{S}_{\max}$ that has the minimum sum of Levenshtein distance to the copies in the cluster.
   end if

1.3 Simulations

We evaluated the accuracy and efficiency of Algorithm 1 by the following simulations. These simulations were tested over sequences of length $n = 200$, clusters of size $4 \leqslant t \leqslant 10$, and deletion probability p in the range $[0.01, 0.10]$. The alphabet size was 4. Each simulation consisted of 100,000 randomly generated clusters. Furthermore, we had another set of simulations for $n = 100$ with deletion probability p in the range $[0.11, 0.20]$ and clusters of size $4 \leqslant t \leqslant 10$. Each simulation for these values of p,n, and t included 10,000

Algorithm 2 ML-Supersequence

Input:

- Cluster $\mathbf{C}$ of t noisy traces: $\boldsymbol{y}_1, \boldsymbol{y}_2, \ldots, \boldsymbol{y}_t$.
- $\mathbf{S}$=$\{\boldsymbol{s}_1, \boldsymbol{s}_2, \ldots, \boldsymbol{s}_k\}$, a set of k candidates.

Output:
Maximum likelihood candidate of $\mathbf{S}$, that is supersequence of all copies from the cluster. If it is not exists, the algorithm returns ϵ.

1. Filter $\mathbf{S}$ so it contains only sequences which are supersequence of all traces from the cluster.
2. **if** $S \neq \emptyset$ **then**
   Return the maximum likelihood sequence from $\mathbf{S}$ with a respect to cluster $\mathbf{C}$.
   end if
3. Return ϵ.

randomly selected clusters. We calculated the Levenshtein error rate (LER) of the decoded output sequence as well as the average decoding success probability (referred as the *success rate*). We also calculated the k-*error success rate*, which is defined as the fraction of clusters where the Levenshtein distance between the algorithm's output sequence and the original sequence was at most k. Note that for $k = 0$, this is equivalent to calculate the success rate. We also calculated the minimal k for which its k-error success rate is at least q, and denote this value of k by k_{q_succ}. Note that for $q = 1$ this value determines the minimal number of Levenshtein errors that an error- correcting code must correct in order to fully decode the original sequences using Algorithm 1 with an error-correcting code. In addition, each cluster was also reconstructed using the BMA algorithm [2].

Figure S1 presents the LER as computed in our simulations of Algorithm 1 and the BMA algorithm for clusters of sizes $t = 7$ and $t = 10$. We also added the trivial lower bound of p^t on the LER [8, 9]. This bound corresponds to the case when the same symbol is deleted in all of the traces. In this case, this symbol will not appear in the list of SCSs of any possible r-tuple or even the entire cluster since it cannot be recovered. Hence, it is not possible to recover its value and thus it will be deleted also in the output of the ML decoder.

In order to simulate also high deletion probabilities, we simulated 1000 clusters of sequences over 4-ry alphabet of length $n = 100$ with cluster size t between 4 and 10, while the deletion probability was $p = 0.25$. Figure S2(a) presents the k-error success rate of this simulation and Figure S2(b) presents the values of k_{1_succ} and $k_{0.99\_\text{succ}}$ by the cluster size in the simulation.

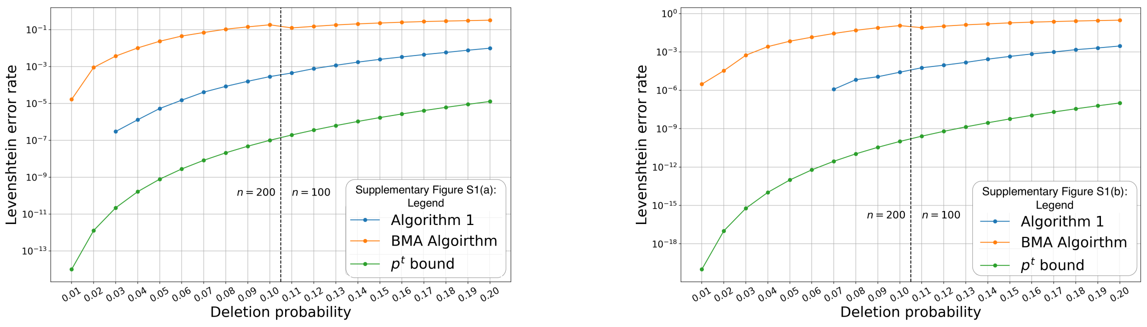

(a) $t = 7$ (b) $t = 10$

Supplementary Figure S1: Levenshtein error rate by the deletion probability p, for clusters of size 7 (left) and 10 (right). This figure presents results from Algorithm 1, the BMA algorithm [2], and the p^t lower bound. Note that the LER was 0 for $p \leqslant 0.06$ and $p \leqslant 0.03$ for $t = 10$ and $t = 7$, respectively. The X-axis represents the different values of the deletion probability in the range $[0.01, 0.20]$ and the Y-axis represents the average LER of the clusters.

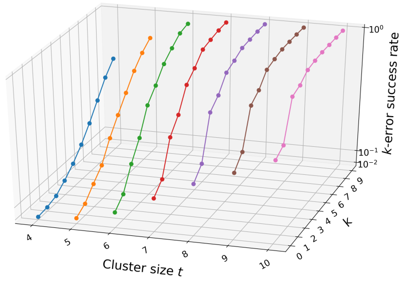

(a) k-error success rate by cluster size t and different values of k. The X-axis represents the cluster size t, the Y-axis represents the value of k for the calculation of the k-error success rate, and the Z-axis represents the k-error success rate.

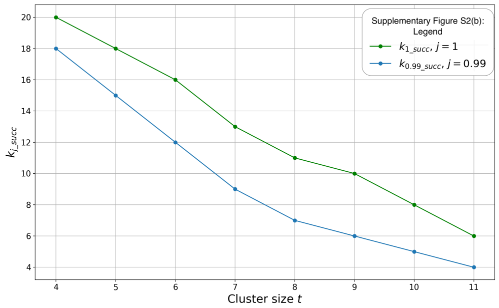

(b) The values of k_{1_succ} and $k_{0.99\_\mathrm{succ}}$ by cluster size t. Denote that, k_{1_succ} is the minimal Levenshtein errors, that an error correcting code must correct in order to fully reconstruct the tested clusters using Algorithm 1. The X-axis represents the cluster size t, the Y-axis represents the k_{j_succ}.

Supplementary Figure S2: k-error success rate, k_{1_succ} and k_{j_succ} values by the cluster size $4 \leqslant t \leqslant 10$. The deletion probability was 0.25.

1.4 Large Cluster

In case the cluster is of larger size, for example in the order of $\Theta(n)$, we present in Algorithm 3, a variation of Algorithm 1 for large clusters. In this case, since the cluster is large, the probability to find a pair, triplet, or quadruplet of traces that their set of SCSs contains the original sequence $\boldsymbol{x}$ is very high, if not even 1. In fact, in all of our simulations, which we will elaborate below in this section, we were always able to successfully decode the original sequence with no errors even when the deletion probability was as high as 0.2. Hence, our main goal in this part is to decrease the runtime of Algorithm 1 while preserving the success rate to be 1. Algorithm 3 keeps the same structure of Algorithm 1, however, it performs two filters on the cluster in order to reduce the computation time.

The complexity of finding the length of the SCS of some set of r traces is the multiplication of their lengths, i.e., $\Theta(n^r)$ [5]. Therefore, the complexity of finding the length of the SCS of a pair of traces is $\Theta(n^2)$, while there are $\Theta(n^2)$ pairs of traces (assuming the cluster size is $\Theta(n)$). Therefore, in this case, calculating the length of the SCS of each pair of traces before considering some triplets is not necessarily the right strategy when our goal is to optimize the algorithm's running time. Hence, in Algorithm 3 we focused on filtering the traces in the cluster in order to check only a subset of the traces which are more likely to succeed and produce the correct sequence.

To define the filtering criteria for Algorithm 3, we simulated Algorithm 1 on large clusters. The length of the original sequence $\boldsymbol{x}$ was $n = 200$ and the cluster size was $t = \frac{n}{2} = 100$. We generated 10,000 clusters of size t, where the deletion probability p was in the range $[0.01, 0.15]$. The success rate of all the simulations was 1. We evaluated the percentage of clusters that the first r-tuple to have an SCS of length n was consisted of the longest 20% traces in the cluster. We observed that when the deletion probability was at most 0.07, in all of the clusters the first r-tuple of traces that had an SCS of size n consisted from the longest 20% traces in the cluster. For deletion probabilities between 0.08 and 0.11 these percentages ranged between 94.76% and 99.98%, while for $p = 0.15$ this percentage was 60.88%. Therefore, by filtering the longest 20% traces, it was enough to check only $\binom{20}{2}$ pairs instead of $\binom{100}{2}$ pairs in order to succeed and still reach the successful pair. The results of these simulations are depicted in Figure S4(a).

This observation lead us to the first filter in Algorithm 3, where we picked the longest 20% traces of the cluster. The second filter computes a cost function (in linear time complexity), to be explained below, on a given r-tuple of traces in order to evaluate if the traces in this r-tuple are likely to have an SCS of length n. Thus, the algorithm skips on the SCS computation of r-tuples that are less likely to have an SCS of length n. First, before performing the first filter, the algorithm calculates the average length of the traces in the cluster and uses it to estimate the deletion probability p. Then, if $p > 0.1$, the algorithm calculates the cost function on every r-tuple and checks if it is higher than some fixed threshold. This threshold depends on the estimated value of p and the cost function is based on a characterization of the sequences, as will be described in Section 1.4.2.

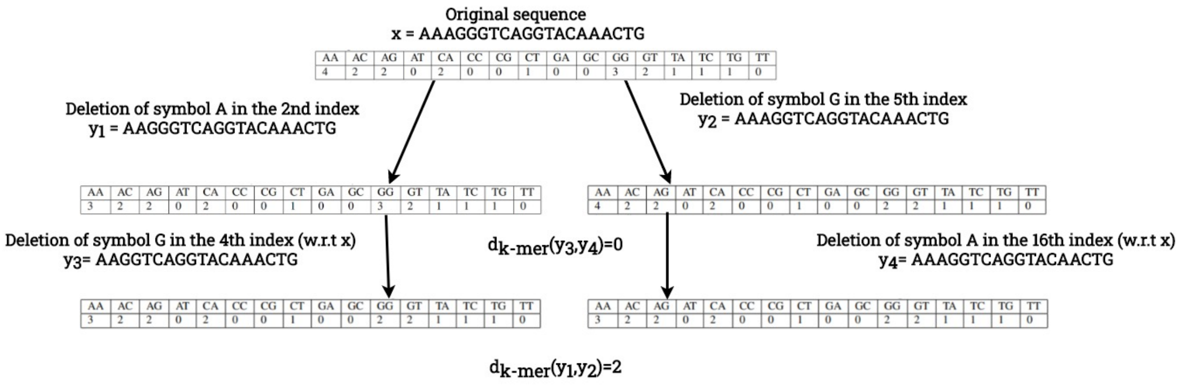

Supplementary Figure S3: k-mer distance demonstration for 4 traces. The original strand $\boldsymbol{x}$ is of length $n = 20$.

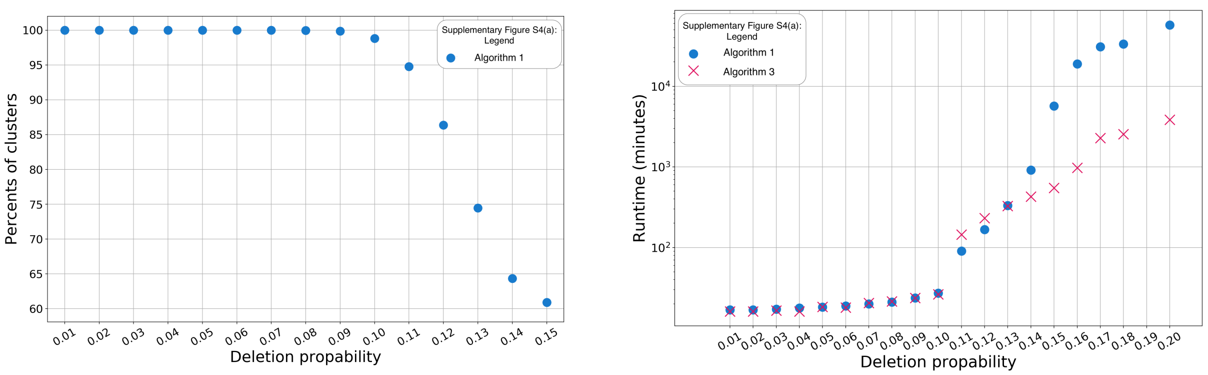

(a) Percents of clusters that the r-tuple of traces that was used by Algorithm 1 to reconstruct the original sequence $\boldsymbol{x}$, was consisted of the longest 20% traces. The X-axis presents the deletion probability and the Y-axis presents the percents of the 10,000 clusters that satisfied this property.

(b) Running time in minutes of performing Algorithm 1 and Algorithm 3 on 10,000 clusters. The X-axis presents the deletion probability and the Y-axis presents the running time in minutes of performing Algorithm 1 and Algorithm 3 on 10,000 clusters of size $t = 100$.

Supplementary Figure S4: Performance evaluation of Algorithm 1 and Algorithm 3. The simulation were for clusters of size $t = 100$, design length of $n = 200$, for each probability p we simulate 10,000 clusters. In all of the simulations the original sequence was reconstructed by the algorithms, introducing a success rate of 1.

1.4.1 An Algorithm for Large Values of t

In this section we present Algorithm 3. We list here the steps that are different from Algorithm 1. In Step 2 the algorithm estimates the deletion probability in the cluster by checking the average length of the traces n' and then calculates $p = 1 - \frac{n'}{n}$. In Step 3, the algorithm filters the cluster so it contains only the longest 20% traces. The last difference between Algorithm 3 and Algorithm 1 can be found in Step 4-b. In this step, before the computation of the SCS of a given r-tuple of traces, the algorithm computes the k-mer cost function (for k-mers of size $k = 2$) and checks if it is larger than the threshold T_p.

We evaluated the performance of Algorithm 3 and verified our filters by simulations. Each simulation consisted of 10,000 clusters of size $t = 100$, the length of the original strand was $n = 200$, the alphabet size was $q = 4$, and the deletion probability p was in the range $[0.01, 0.2]$. Algorithm 3 reconstructed the exact sequence $\boldsymbol{x}$ in all of the tested clusters. A comparison between the runtime of Algorithm 1 and Algorithm 3 can be found in Figure S4(b). Note that we did not compare the running time with the BMA algorithm since its success rate was significantly lower, for example when the deletion probability was 15%, its success rate was roughly 0.46.

Algorithm 3 ML-SCS Reconstruction for Large Clusters

Input:

- Cluster $\mathbf{C}$ of $t = \Theta(n)$ noisy traces: $\boldsymbol{y}_1, \boldsymbol{y}_2, \ldots, \boldsymbol{y}_t$ sorted by their lengths in a non-decreasing order.
- Design length= n.

Output: $\widehat{\boldsymbol{x}}$ - Estimation of the original sequence.

1. $\widehat{\boldsymbol{x}} = \epsilon$, $n_{\max} = 0$, $\mathbf{C}_{\max} = \emptyset$.
2. Compute n' the mean length of the traces in $\mathbf{C}$, and define $p = 1 - \frac{n'}{n}$.
3. Filter traces from $\mathbf{C}$ so it contains only the $t' = 0.2t$ first traces in the cluster.
4. **for** r = 2, 3, 4 **do**
   (a) Denote F=$\{\boldsymbol{c}_i^{(r)} = (\boldsymbol{y}_{i_1}, \boldsymbol{y}_{i_2}, \ldots, \boldsymbol{y}_{i_r}) | 1 \leqslant i \leqslant \binom{t'}{r}, 1 \leqslant i_1 < i_2 < \cdots < i_r \leqslant t'\}$ the set of all r-tuples from $\mathbf{C}$, sorted by non-decreasing order of the sum of the lengths of the copies in each tuple.
   (b) **for** i= 1, 2, ..., $\binom{t'}{r}$ **do**
      if $p > 0.1$ and $c_{k\text{-mer}}(\boldsymbol{c}_i^{(r)}) > 0.25np(2k-1)$ **then**
         /* k-mer size $k = 2$. */
         if $\mathsf{SCS}(\boldsymbol{c}_i^{(r)}) = n$ **then**
            $\mathbf{S} = \mathcal{S}\mathsf{CS}(\boldsymbol{c}_i^{(r)})$
            $\widehat{\boldsymbol{x}}$= ML-Supersequence($\mathbf{S}, \mathbf{C}$)
            if $\widehat{\boldsymbol{x}} \neq \epsilon$ **then**
               return $\widehat{\boldsymbol{x}}$
            end if
         else
            if $\mathsf{SCS}(\boldsymbol{c}_i^{(r)}) > n_{\max}$ **then**
               $n_{\max} = \mathsf{SCS}(\boldsymbol{c}_i^{(r)})$
               $\mathbf{C}_{\max} = \mathbf{C}_{\max} \cup \{\boldsymbol{c}_i^{(r)}\}$
            end if
            if $\mathsf{SCS}(\boldsymbol{c}_i^{(r)}) = n_{\max}$ **then**
               $\mathbf{C}_{\max} = \mathbf{C}_{\max} \cup \{\boldsymbol{c}_i^{(r)}\}$
            end if
         end if
      end if
   (c) **end for**
   end for
5. Compute $\mathbf{S}_{\max}$=$\bigcup_{\boldsymbol{c} \in \mathbf{C}_{\max}} \mathcal{S}\mathsf{CS}(\boldsymbol{c})$, the union of all $\mathcal{S}\mathsf{CS}$ of $\boldsymbol{c}_i^{(r)} \in \mathbf{C}_{\max}$.
6. $\widehat{\boldsymbol{x}}$=ML-Supersequence($\mathbf{S}_{\max}, \mathbf{C}$)
7. **if** $\widehat{\boldsymbol{x}} \neq \epsilon$ **then**
      return $\widehat{\boldsymbol{x}}$
   else
      Return the sequence from $\mathbf{S}_{\max}$ that has the minimum sum of Levenshtein distance to the copies in the cluster.
   end if

1.4.2 The k-mer Distance and the k-mer Cost Function

The *k-mer vector* of a sequence $\boldsymbol{y}$, denoted by k-mer($\boldsymbol{y}$), is a vector that counts the frequency in $\boldsymbol{y}$ of each subsequence of length k (k-mer). The frequencies are ordered in a lexicographical order of their corresponding k-mers. For example for a given sequence $\boldsymbol{y}$ = "$ACCTCC$" and $k = 2$, its k-mer vector is k-mer($\boldsymbol{y}$) = 0100020100000101, according to the following calculation of the frequencies $\{AA : 0, AC : 1, AG : 0, AT : 0, CA : 0, CC : 2, CG : 0, CT : 1, GA : 0, GC : 0, GG : 0, GT : 0, TA : 0, TC : 1, TG : 0, TT : 1\}$. We define the *$k$-mer distance* between two sequences $\boldsymbol{y}_1$ and $\boldsymbol{y}_2$ as the L_1 distance between their k-mer vectors. The k-mer distance is denoted by $d_{k\text{-mer}}(\boldsymbol{y}_1, \boldsymbol{y}_2)$.

$$d_{k\text{-mer}}(\boldsymbol{y}_1, \boldsymbol{y}_2) = ||\boldsymbol{y}_1 - \boldsymbol{y}_2||_1.$$

For a given set of r sequences $\mathbf{Y} = \{\boldsymbol{y}_1, \boldsymbol{y}_2, \dots, \boldsymbol{y}_r\}$, we define its *$k$-mer cost function*, which is denoted by $c_{k\text{-mer}}(\boldsymbol{y}_1, \boldsymbol{y}_2, \dots, \boldsymbol{y}_r)$, as the sum of the k-mer distance of each pair of sequences in $\mathbf{Y}$. That is,

$$c_{k\text{-mer}}(\boldsymbol{y}_1, \boldsymbol{y}_2, \dots, \boldsymbol{y}_r) = \sum_{1 \leqslant i < j \leqslant r} d_{k\text{-mer}}(\boldsymbol{y}_i, \boldsymbol{y}_j).$$

Observe that the k-mer distance between a sequence $\boldsymbol{x}$ and a trace $\boldsymbol{y}_1$ which results from $\boldsymbol{x}$ by one deletion is at most $2k - 1$. Every deleted symbol in $\boldsymbol{x}$ decreases the value of at most k entries in k-mer($\boldsymbol{x}$) and increases the number of at most $k - 1$ of the entries. Hence, each deletion increases the k-mer distance by at most $2k - 1$, which means that an upper bound on the k-mer distance between the original strand $\boldsymbol{x}$ and a trace $\boldsymbol{y}_i$ with np deletions is $np(2k - 1)$. However, when comparing the k-mer distance of two traces, $\boldsymbol{y}_1$ and $\boldsymbol{y}_2$, with more than one deletion, the k-mer distance can also decrease. An example of such a case is depicted in Figure 3. Combining these two observations, Algorithm 3 estimates if two traces have relatively large Levenshtein distance. If these traces have large Levenshtein distance, it is more likely that both of them will have an SCS of length n. Hence, the algorithm checks if the k-mer distance is larger than the threshold $T_p = 0.25np(2k - 1)$ and continues to compute the SCS, only if the condition holds. A similar computation is done for tuples with more than two traces. We use the value of 0.25 in the threshold to consider the cases where the k-mer distance decreases as depicted in Figure S3. We selected this value after simulating other values as well, reaching the best result with 0.25. An optimization of this value can be done in further research.

2 The DNA Reconstruction Problem

This section studies the DNA reconstruction problem. Assume that a cluster consists of t traces, $\boldsymbol{y}_1, \boldsymbol{y}_2, \dots, \boldsymbol{y}_t$, where all of them are noisy copies of a synthesized strand. This model assumes that every trace is a sequence that is independently received by the transmission of a length-n sequence $\boldsymbol{x}$ (the synthesized strand) through a deletion-insertion-substitution channel with some fixed probability p_d for deletion, p_i for insertion, and p_s for substitution. Our goal is to propose an efficient algorithm which returns $\widehat{\boldsymbol{x}}$, an estimation of the transmitted sequence $\boldsymbol{x}$, with the intention of minimizing the edit distance between $\boldsymbol{x}$ and $\widehat{\boldsymbol{x}}$. In our simulations, we consider several values of t and a wide range of error probabilities as well as data from previous DNA storage experiments.

Before we present the algorithms, we list here several more notations and definitions. An *error vector* of $\boldsymbol{y}$ and $\boldsymbol{x}$, denoted by $EV(\boldsymbol{y}, \boldsymbol{x})$, is a vector of minimum number of edit operations to transform $\boldsymbol{y}$ to $\boldsymbol{x}$. Each entry in $EV(\boldsymbol{y}, \boldsymbol{x})$ consists of the index in $\boldsymbol{y}$, the original symbol in this index, the edit operation and in case the operation is an insertion, substitution the entry also includes the inserted, substituted symbol, respectively. Note that for two sequences $\boldsymbol{y}$ and $\boldsymbol{x}$, there could be more than one sequence of edit operations to transform $\boldsymbol{y}$ to $\boldsymbol{x}$. The edit distance between a pair of sequences is computed using a dynamic programming table and the error vector is computed by backtracking on this table. Hence, $EV(\boldsymbol{y}, \boldsymbol{x})$ is not unique and can be defined uniquely by giving priorities to the different operation in case of ambiguity. That is, if there is an entry in the vector $EV(\boldsymbol{y}, \boldsymbol{x})$ (from the last entry to the first), where more than one edit operation can be selected, then, the operation is selected according to these given priorities. The error vector $EV(\boldsymbol{y}, \boldsymbol{x})$ also maps each symbol in $\boldsymbol{y}$ to a symbol in $\boldsymbol{x}$ (and vice versa). We denote this mapping as $V_{EV}(\boldsymbol{y}, \boldsymbol{x}) : \{1, 2, \dots, |\boldsymbol{y}|\} \to \{1, 2, \dots, |\boldsymbol{x}|\} \cup \{?\}$, where $V_{EV}(\boldsymbol{y}, \boldsymbol{x})(i) = j$ if and only if the i-th symbol in $\boldsymbol{y}$ appears as the j-th symbol in $\boldsymbol{x}$, with respect to the error vector $EV(\boldsymbol{y}, \boldsymbol{x})$. Note that in the case where the i-th symbol in $\boldsymbol{y}$ was classified as a deleted symbol in $EV(\boldsymbol{y}, \boldsymbol{x})$, $V_{EV}(\boldsymbol{y}, \boldsymbol{x})(i) =?$. This mapping can also be represented as a vector of size $|\boldsymbol{y}|$, where the i-th entry in this vector is $V_{EV}(\boldsymbol{y}, \boldsymbol{x})(i)$. The *reversed*

cluster of a cluster $\mathbf{C}$, denoted by $\mathbf{C}^R$, consists of the traces in $\mathbf{C}$ where each one of them is reversed. The reverse trace of $\boldsymbol{y}_i \in \mathbf{C}$ is denoted by $\boldsymbol{y}_i^R \in \mathbf{C}$. The symbols of each trace $\boldsymbol{y}_i^R \in \mathbf{C}^R$ are arranged in reverse order compared to how they appear in their original form $\boldsymbol{y}_i \in \mathbf{C}$. For example, for cluster $\mathbf{C} = \{\boldsymbol{y}_1 = ACGTC, \boldsymbol{y}_2 = CCGTA\}$, the reversed cluster is $\mathbf{C}^R = \{\boldsymbol{y}_1^R = CTGCA, \boldsymbol{y}_2^R = ATGCC\}$.

2.1 The Iterative Reconstruction Algorithm (ITR Algorithm)

In this section we present Algorithm 4, the *ITR Algorithm*. The algorithm receives a cluster of t traces $\mathbf{C}$ and the design length n. Algorithm 4 uses several methods to revise the traces from the cluster and to generate from the revised traces a multiset of candidates. Then, Algorithm 4 returns the candidate that is most likely to be the original sequence $\boldsymbol{x}$. The methods used to revise the traces are described in this section as Algorithm 5 and Algorithm 6. Algorithm 4 invokes Algorithm 5 and Algorithm 6 on the cluster in two different procedures as described in Algorithm 7 and Algorithm 8.

The first method is described in Algorithm 5 is the EV Algorithm. The algorithm receives $\mathbf{C}$, a cluster of t traces, the design length n, and $\boldsymbol{y}_i$, a trace from the cluster. Algorithm 5 calculates for every $1 \leqslant k \leqslant t, k \neq i$, the vector $EV(\boldsymbol{y}_i, \boldsymbol{y}_k)$. In some fo the cases, there may be more than one error vector for $EV(\boldsymbol{y}_i, \boldsymbol{y}_k)$, which corresponds to the edit operations to transform $\boldsymbol{y}_i$ to $\boldsymbol{y}_k$. In these cases, the algorithm prioritizes substitutions, then insertions, then deletions in order to choose one unique vector. These priorities were selected to support our definition of the deletion-insertion-substitution channel. However, for practical uses, one can easily change these priorities if some preliminary knowledge of the error rates in the data is given. Following that, the algorithm performs a majority vote in each index on these vectors and creates $\mathbf{S}$, which is a vector of edit operations. Lastly, Algorithm 5 performs the edit operations on $\boldsymbol{y}_i$, and returns it as an output for Algorithm 7 and Algorithm 8. Algorithm 5 is used as a procedure in Algorithm 7 and Algorithm 8 to correct substitution and insertion errors of the traces in the cluster.

The second method is described in Algorithm 6, is known as the *PP Algorithm*. Similarly to Algorithm 5, Algorithm 6 receives $\mathbf{C}$, a cluster of t traces, the design length n, and $\boldsymbol{y}_k$, a trace from the cluster. Algorithm 6 uses similar patterns (defined in Section 2.1.1) on each pair of traces and creates a weighted graph from them. Each vertex of the graph represents a pattern, and an edge connects patterns with identical prefix and suffix. The weight on each edge represents the frequency of the incoming pattern, the number of pairs of traces in the cluster that have this pattern in their sequences. Algorithm 6 is described in detail in Section 2.1.1. Algorithm 6 is used as a procedure in in Algorithm 7 and Algorithm 8 to correct deletion errors in the traces in the cluster.

Algorithm 7, the *HR algorithm* receives a cluster of t traces $\mathbf{C}$ and the design length n. Algorithm 7 performs k cycles, where in each cycle it iterates over all the traces in the cluster. For each trace $\boldsymbol{y}_k$, it first uses Algorithm 5 to correct substitution errors, then it uses Algorithm 6 to correct deletion errors, and lastly, it uses Algorithm 5 to correct insertion errors. When it finishes iterating over the traces in the cluster, Algorithm 7 updates the cluster with all the revised traces and continues to the next cycle. At the end, Algorithm 7 performs the same procedure on $\mathbf{C}^R$. Algorithm 7 returns a multiset of all the revised traces.

Algorithm 8, the *VR algorithm* also receives a cluster of t traces $\mathbf{C}$ and the design length n. Algorithm 8 uses the same procedures as Algorithm 7. However, in each cycle, it first corrects substitutions in all of the traces in the cluster using algorithm 5, then it invokes algorithm 6 on each trace to correct deletions, and finally invokes Algorithm 5 to correct insertions. Similarly to Algorithm 7, Algorithm 8 performs the same operations also on $\mathbf{C}^R$ and returns a multiset of the results.

Algorithm 4 invokes Algorithms 7 and 8, with $k = 2$ cycles and combines the resulted multisets to the multiset $\mathbf{S}$. If one or more sequences of length n exists in the multiset $\mathbf{S}$, it returns the one that minimizes the sum of edit distances to the traces in the cluster. Otherwise, it checks if there are sequences of length $n-1$ or $n+1$ in $\mathbf{S}$, and returns the most frequent among them. If such a sequence does not exists, it returns the first sequence in $\mathbf{S}$. The number of cycles is $k = 2$ since we have found out that for most of the clusters, the set of candidates converges after two cycles. Thus, a possible third cycle can not improve the results in most of the cases.

2.1.1 The Pattern Path Algorithm (PP Algorithm)

In this section we present the Pattern Path algorithm. The algorithm, described also in Algorithm 6, is the main procedure of the iterative algorithm (ITR Algorithm) that corrects edit errors. Denote by $\boldsymbol{w}$ an arbitrary LCS sequence of $\boldsymbol{x}$ and $\boldsymbol{y}$ of length ℓ. The sequence $\boldsymbol{w}$ is a subsequence of $\boldsymbol{x}$, and hence, all of its ℓ symbols appear in some indices of $\boldsymbol{x}$, and assume these indices are given by $i_1^{\boldsymbol{x}} < i_2^{\boldsymbol{x}} < \cdots < i_\ell^{\boldsymbol{x}}$. It should be

Algorithm 4 Iterative Reconstruction (The ITR Algorithm)

Input:

- Cluster $\mathbf{C}$ of t noisy traces: $\boldsymbol{y}_1, \boldsymbol{y}_2, \ldots, \boldsymbol{y}_t$.
- Design length $= n$.

Output:

- $\widehat{\boldsymbol{x}}$ - Estimation of the original sequence.

1. $\mathbf{S} = \emptyset$
2. Use Algorithm 7 and Algorithm 8, with $\mathbf{C}, n, k = 2$ as parameters, to compute a multiset of candidates. Save the candidates and their frequencies in it in $\mathbf{S}$.
3. If $\mathbf{S}$ has one or more sequence of length n, return one that minimizes the sum of edit distance to the traces in $\mathbf{C}$ (ties are breaking randomly).
4. Otherwise, if $\mathbf{S}$ includes sequences of length $n-1$ or $n+1$ that minimize the sum of edit distance to the traces in $\mathbf{C}$, return the sequence which is most frequent in the multiset $\mathbf{S}$ (if there is more than one choose randomly).
5. Return the first sequence in $\mathbf{S}$.

noted that a subsequence can have more than one set of such indices, while the number of such sets is defined as the embedding number [1, 3]. In our algorithm, we chose one of these sets arbitrarily. Furthermore, given a set of such indices $i_1^{\boldsymbol{x}} < i_2^{\boldsymbol{x}} < \cdots < i_\ell^{\boldsymbol{x}}$, we define the *embedding sequence* of $\boldsymbol{w}$ in $\boldsymbol{x}$, denoted by $\boldsymbol{u}_{\boldsymbol{x},\boldsymbol{w}}$, as a sequence of length $|\boldsymbol{x}|$ where for $1 \leqslant j \leqslant \ell$, $\boldsymbol{u}_{\boldsymbol{x},\boldsymbol{w}}(i_j^{\boldsymbol{x}})$ equals to $\boldsymbol{x}(i_j^{\boldsymbol{x}})$ and otherwise it equals to ?.

The *gap* of $\boldsymbol{x}$, $\boldsymbol{y}$ and their length-ℓ LCS sequence $\boldsymbol{w}$ in index $1 \leqslant j \leqslant |\boldsymbol{x}|$ with respect to $\boldsymbol{u}_{\boldsymbol{x},\boldsymbol{w}}$ and $\boldsymbol{u}_{\boldsymbol{y},\boldsymbol{w}}$, denoted by $\text{gap}_{\boldsymbol{u}_{\boldsymbol{x},\boldsymbol{w}}}^{\boldsymbol{u}_{\boldsymbol{y},\boldsymbol{w}}}(j)$, is defined as follows. In case the j-th or the $(j-1)$-th symbol in $\boldsymbol{u}_{\boldsymbol{x},\boldsymbol{w}}$ equals ?, $\text{gap}_{\boldsymbol{u}_{\boldsymbol{x},\boldsymbol{w}}}^{\boldsymbol{u}_{\boldsymbol{y},\boldsymbol{w}}}(j)$ is defined as an empty sequence. Otherwise, the symbol $\boldsymbol{u}_{\boldsymbol{x},\boldsymbol{w}}(j)$ also appears in $\boldsymbol{w}$. Denote by j', the index of the symbol $\boldsymbol{u}_{\boldsymbol{x},\boldsymbol{w}}(j)$ in $\boldsymbol{w}$. Recall that the sequence $\boldsymbol{w}$ is an LCS of $\boldsymbol{x}$ and $\boldsymbol{y}$, and $\boldsymbol{u}_{\boldsymbol{y},\boldsymbol{w}}$ is the embedding sequence of $\boldsymbol{w}$ in $\boldsymbol{y}$. Given $\boldsymbol{u}_{\boldsymbol{y},\boldsymbol{w}}$, we can define the sequence of indices $i_1^{\boldsymbol{y}} < i_2^{\boldsymbol{y}} < \cdots < i_\ell^{\boldsymbol{y}}$, such that $\boldsymbol{w}(j') = \boldsymbol{y}(i_{j'}^{\boldsymbol{y}})$ for $1 \leqslant j' \leqslant \ell$. Given such a sequence of indices, $\text{gap}_{\boldsymbol{u}_{\boldsymbol{x},\boldsymbol{w}}}^{\boldsymbol{u}_{\boldsymbol{y},\boldsymbol{w}}}(j)$ is defined as the sequence $\boldsymbol{y}_{[i_{j'-1}^{\boldsymbol{y}}+1:i_{j'}^{\boldsymbol{y}}-1]}$, which is the sequence between the appearances of the j'-th and the $(j'-1)$-th symbols of $\boldsymbol{w}$ in $\boldsymbol{y}$. Note that since $i_{j'}^{\boldsymbol{y}}$ can be equal to $i_{j'-1}^{\boldsymbol{y}}+1$, $\text{gap}_{\boldsymbol{u}_{\boldsymbol{x},\boldsymbol{w}}}^{\boldsymbol{u}_{\boldsymbol{y},\boldsymbol{w}}}(j)$ can be an empty sequence. Roughly speaking, the $\text{gap}_{\boldsymbol{u}_{\boldsymbol{x},\boldsymbol{w}}}^{\boldsymbol{u}_{\boldsymbol{y},\boldsymbol{w}}}(j)$ holds every symbol that appears in $\boldsymbol{y}$ between the $(j'-1)$-th and j'-th symbols of the LCS $\boldsymbol{w}$, based on the embedding sequence $\boldsymbol{u}_{\boldsymbol{w},\boldsymbol{y}}$.

The *pattern* of $\boldsymbol{x}$ and $\boldsymbol{y}$ with respect to the LCS sequence $\boldsymbol{w}$, its embedding sequences $\boldsymbol{u}_{\boldsymbol{x},\boldsymbol{w}}$ and $\boldsymbol{u}_{\boldsymbol{y},\boldsymbol{w}}$, an index $1 \leqslant i \leqslant |\boldsymbol{x}|$ and a length $m \geqslant 2$, denoted by $Ptn(\boldsymbol{x}, \boldsymbol{y}, \boldsymbol{w}, \boldsymbol{u}_{\boldsymbol{x},\boldsymbol{w}}, \boldsymbol{u}_{\boldsymbol{y},\boldsymbol{w}}, i, m)$, is defined as:

$$Ptn(\boldsymbol{x}, \boldsymbol{y}, \boldsymbol{w}, \boldsymbol{u}_{\boldsymbol{x},\boldsymbol{w}}, \boldsymbol{u}_{\boldsymbol{y},\boldsymbol{w}}, i, m) \triangleq (\boldsymbol{u}_{\boldsymbol{x},\boldsymbol{w}}(i-1), \text{gap}_{\boldsymbol{u}_{\boldsymbol{x},\boldsymbol{w}}}^{\boldsymbol{u}_{\boldsymbol{y},\boldsymbol{w}}}(i), \boldsymbol{u}_{\boldsymbol{x},\boldsymbol{w}}(i), \ldots, \text{gap}_{\boldsymbol{u}_{\boldsymbol{x},\boldsymbol{w}}}^{\boldsymbol{u}_{\boldsymbol{y},\boldsymbol{w}}}(i+m-1), \boldsymbol{u}_{\boldsymbol{x},\boldsymbol{w}}(i+m-1)),$$

where for $i < 1$ and $i > |\boldsymbol{x}|$, the symbol $\boldsymbol{u}_{\boldsymbol{x},\boldsymbol{w}}(i)$ is defined as the null character and $\text{gap}_{\boldsymbol{u}_{\boldsymbol{x},\boldsymbol{w}}}^{\boldsymbol{u}_{\boldsymbol{y},\boldsymbol{w}}}(i)$ is defined as an empty sequence. The parameter m defines the length of the pattern, that is the number of embedding sequences and gaps that comprises the patterns. In our implementation of the algorithm, the length of the patterns is defined as $m = 2$.

We also define the prefix and suffix of a pattern $Ptn(\boldsymbol{x}, \boldsymbol{y}, \boldsymbol{w}, \boldsymbol{u}_{\boldsymbol{x},\boldsymbol{w}}, \boldsymbol{u}_{\boldsymbol{y},\boldsymbol{w}}, i, m)$ to be:

$$\text{Prefix}(Ptn(\boldsymbol{x}, \boldsymbol{y}, \boldsymbol{w}, \boldsymbol{u}_{\boldsymbol{x},\boldsymbol{w}}, \boldsymbol{u}_{\boldsymbol{y},\boldsymbol{w}}, i, m)) \triangleq (\boldsymbol{u}_{\boldsymbol{x},\boldsymbol{w}}(i-1), \text{gap}_{\boldsymbol{u}_{\boldsymbol{x},\boldsymbol{w}}}^{\boldsymbol{u}_{\boldsymbol{y},\boldsymbol{w}}}(i), \boldsymbol{u}_{\boldsymbol{x},\boldsymbol{w}}(i), \ldots, \boldsymbol{u}_{\boldsymbol{x},\boldsymbol{w}}(i+m-2)),$$

$$\text{Suffix}(Ptn(\boldsymbol{x}, \boldsymbol{y}, \boldsymbol{w}, \boldsymbol{u}_{\boldsymbol{x},\boldsymbol{w}}, \boldsymbol{u}_{\boldsymbol{y},\boldsymbol{w}}, i, m) \triangleq (\boldsymbol{u}_{\boldsymbol{x},\boldsymbol{w}}(i), \text{gap}_{\boldsymbol{u}_{\boldsymbol{x},\boldsymbol{w}}}^{\boldsymbol{u}_{\boldsymbol{y},\boldsymbol{w}}}(i+1), \ldots, \boldsymbol{u}_{\boldsymbol{x},\boldsymbol{w}}(i+m-1)).$$

Finally, we define

$$P(\boldsymbol{x}, \boldsymbol{y}, \boldsymbol{w}, \boldsymbol{u}_{\boldsymbol{x},\boldsymbol{w}}, \boldsymbol{u}_{\boldsymbol{y},\boldsymbol{w}}, m) \triangleq \{Ptn(\boldsymbol{x}, \boldsymbol{y}, \boldsymbol{w}, \boldsymbol{u}_{\boldsymbol{x},\boldsymbol{w}}, \boldsymbol{u}_{\boldsymbol{y},\boldsymbol{w}}, , i, m) : 1 \leqslant i \leqslant |\boldsymbol{x}|\}.$$

The Pattern Path Algorithm receives a cluster $\mathbf{C}$ of t traces and one of the traces in the cluster $\boldsymbol{y}_k$. First, the algorithm initializes $L[\boldsymbol{y}_k]$, which is a set of $|\boldsymbol{y}_k|$ empty lists. For $1 \leqslant i \leqslant |\boldsymbol{y}_k|$, the i-th list of $L[\boldsymbol{y}_k]$ is denoted by $L[\boldsymbol{y}_k]_i$. The algorithm pairs $\boldsymbol{y}_k$ with each of the other traces in $\mathbf{C}$. For each pair of traces, $\boldsymbol{y}_k$ and $\boldsymbol{y}_h$, the algorithm computes an arbitrary LCS sequence $\boldsymbol{w}$, and an arbitrary embedding sequence $\boldsymbol{u}_{\boldsymbol{y}_k,\boldsymbol{w}}$. Then it uses $\boldsymbol{w}$ and $\boldsymbol{u}_{\boldsymbol{y}_k,\boldsymbol{w}}$ to compute $P(\boldsymbol{y}_k, \boldsymbol{y}_h, \boldsymbol{w}, \boldsymbol{u}_{\boldsymbol{y}_k,\boldsymbol{w}}, \boldsymbol{u}_{\boldsymbol{y}_h,\boldsymbol{w}}, m)$. For $1 \leqslant i \leqslant |\boldsymbol{y}_k|$, the algorithm saves $Ptn(\boldsymbol{y}_k, \boldsymbol{y}_h, \boldsymbol{w}, \boldsymbol{u}_{\boldsymbol{y}_k,\boldsymbol{w}}, \boldsymbol{u}_{\boldsymbol{y}_h,\boldsymbol{w}}, i, m)$ in $L[\boldsymbol{y}_k]_i$. Then, the algorithm builds the *pattern graph* $G_{pat}(\boldsymbol{y}_k) = (V(\boldsymbol{y}_k), E(\boldsymbol{y}_k))$, which is a directed acyclic graph, and is defined as follows.

1. $V(\boldsymbol{y}_k) = \{((Ptn(\boldsymbol{y}_k, \boldsymbol{y}_h, \boldsymbol{w}, \boldsymbol{u}_{\boldsymbol{y}_k,\boldsymbol{w}}, \boldsymbol{u}_{\boldsymbol{y}_h,\boldsymbol{w}}, i, m), i) : 1 \leqslant h \leqslant t, h \neq k, 1 \leqslant i \leqslant |\boldsymbol{y}_k|\} \cup \{S, U\}$.

   The vertices are pairs of patterns and their index. Note that the same pattern can appear in several vertices with different indices i. The value $|V|$ equals to the number of distinct pattern-index pairs.

2. $E(\boldsymbol{y}_k)=\{e=(v,u): v=(Ptn(\boldsymbol{y}_k, \boldsymbol{y}_h, \boldsymbol{w}, \boldsymbol{u}_{\boldsymbol{y}_k,\boldsymbol{w}}, \boldsymbol{u}_{\boldsymbol{y}_h,\boldsymbol{w}}, i, m), i), u=(Ptn(\boldsymbol{y}_k, \boldsymbol{y}_h, \boldsymbol{w}, \boldsymbol{u}_{\boldsymbol{y}_k,\boldsymbol{w}}, \boldsymbol{u}_{\boldsymbol{y}_h,\boldsymbol{w}}, i{+}1, m), i{+}1),$

   $\text{Suffix}(Ptn(\boldsymbol{y}_k, \boldsymbol{y}_h, \boldsymbol{w}, \boldsymbol{u}_{\boldsymbol{y}_k,\boldsymbol{w}}, \boldsymbol{u}_{\boldsymbol{y}_h,\boldsymbol{w}}, i, m) = \text{Preffix}(Ptn(\boldsymbol{y}_k, \boldsymbol{y}_h, \boldsymbol{w}, \boldsymbol{u}_{\boldsymbol{y}_k,\boldsymbol{w}}, \boldsymbol{u}_{\boldsymbol{y}_h,\boldsymbol{w}}, i{+}1, m)\}$.

3. The weights of the edges are defined by $w : E \to N$ as follows:

   For $e = (v, u)$, where $u = (Ptn(\boldsymbol{y}_k, \boldsymbol{y}_h, \boldsymbol{w}, \boldsymbol{u}_{\boldsymbol{y}_k,\boldsymbol{w}}, \boldsymbol{u}_{\boldsymbol{y}_h,\boldsymbol{w}}, i, m), i)$, it holds that

   $$w(e) = |\{Ptn \in L[\boldsymbol{y}_k]_i : Ptn = Ptn(\boldsymbol{y}_k, \boldsymbol{y}_h, \boldsymbol{w}, \boldsymbol{u}_{\boldsymbol{y}_k,\boldsymbol{w}}, \boldsymbol{u}_{\boldsymbol{y}_h,\boldsymbol{w}}, i, m)\}|,$$

   which is the number of appearances of $Ptn(\boldsymbol{y}_k, \boldsymbol{y}_h, \boldsymbol{w}, \boldsymbol{u}_{\boldsymbol{y}_k,\boldsymbol{w}}, \boldsymbol{u}_{\boldsymbol{y}_h,\boldsymbol{w}}, i, m)$ in $L[\boldsymbol{y}_k]_i$.

4. The vertex S, which does not correspond to any pattern, is connected to all vertices of the first index. The weight of these edges is the number of appearances of the incoming vertex pattern.

5. The vertex U has incoming edges from all vertices of the last index and the weight of each edge is zero.

Finally, the Pattern Path Algorithm identifies a longest path from S to U in the graph. This path induces a sequence, denoted by $\widehat{\boldsymbol{y}}_k$, which is formed by concatenating the patterns of $\boldsymbol{y}_k$ (including their gaps if such exist), that appears in the vertices of the longest path in the pattern graph. It's important to note that $\widehat{\boldsymbol{y}}_k$ represents a modified version of $\boldsymbol{y}_k$, as it incorporates the patterns present in $\boldsymbol{y}_k$. The algorithm returns $\widehat{\boldsymbol{y}}_k$, which is an updated rendition of $\boldsymbol{y}_k$, while also incorporating any gaps inherited from the vertices along the longest path. To illustrate the Pattern Path Algorithm's workflow, we provide an example in the following section.

2.2 Example of the PP Algorithm

We present here a short example of the Pattern Path Algorithm and its related definitions.

2.2.1 Input of the algorithm

The original strand in this example is $\boldsymbol{x}$ which is given below. The cluster of its $t = 5$ traces is $\mathbf{C} = \{\boldsymbol{y}_1, \ldots, \boldsymbol{y}_5\}$. The original design length of $\boldsymbol{x}$ is $n = 10$. The traces are noisy copies of $\boldsymbol{x}$ and include deletions, insertions, and substitutions. In this example Algorithm 6 receives the cluster $\mathbf{C}$ and the trace $\boldsymbol{y}_k = \boldsymbol{y}_1$ as its input.

- $\boldsymbol{x}$ = GTAGTGCCTG.
- $\boldsymbol{y}_1$ = GTAGGTGCCG.
- $\boldsymbol{y}_2$ = GTAGTCCTG.
- $\boldsymbol{y}_3$ = GTAGTGCCTG.
- $\boldsymbol{y}_4$ = GTAGCGCCAG.
- $\boldsymbol{y}_5$ = GCATGCTCTG.

2.2.2 Computation of the LCSs and the patterns in cluster

After receiving the input the PP algorithm continues with the next step of computing the LCSs and the patterns of the cluster. Figure S5 presents the process of computing the patterns of $(\boldsymbol{y}_1, \boldsymbol{y}_2), (\boldsymbol{y}_1, \boldsymbol{y}_3), (\boldsymbol{y}_1, \boldsymbol{y}_4), (\boldsymbol{y}_1, \boldsymbol{y}_5)$. For each pair, $\boldsymbol{y}_1$ and $\boldsymbol{y}_i$, Figure S5 depicts $\boldsymbol{w}_i$, which is an LCS of the sequences $\boldsymbol{y}_1$ and $\boldsymbol{y}_i$. Then, the figure presents $\boldsymbol{u}_{\boldsymbol{y}_1,\boldsymbol{w}_i}$ and $\boldsymbol{u}_{\boldsymbol{y}_i,\boldsymbol{w}_i}$, which are the embedding sequences that the Pattern Path Algorithm uses in order to compute the patterns. Lastly, the list of patterns of each pair is depicted in an increasing order of their indices. Note that lowercase symbols present gaps and X presents the symbol ?.

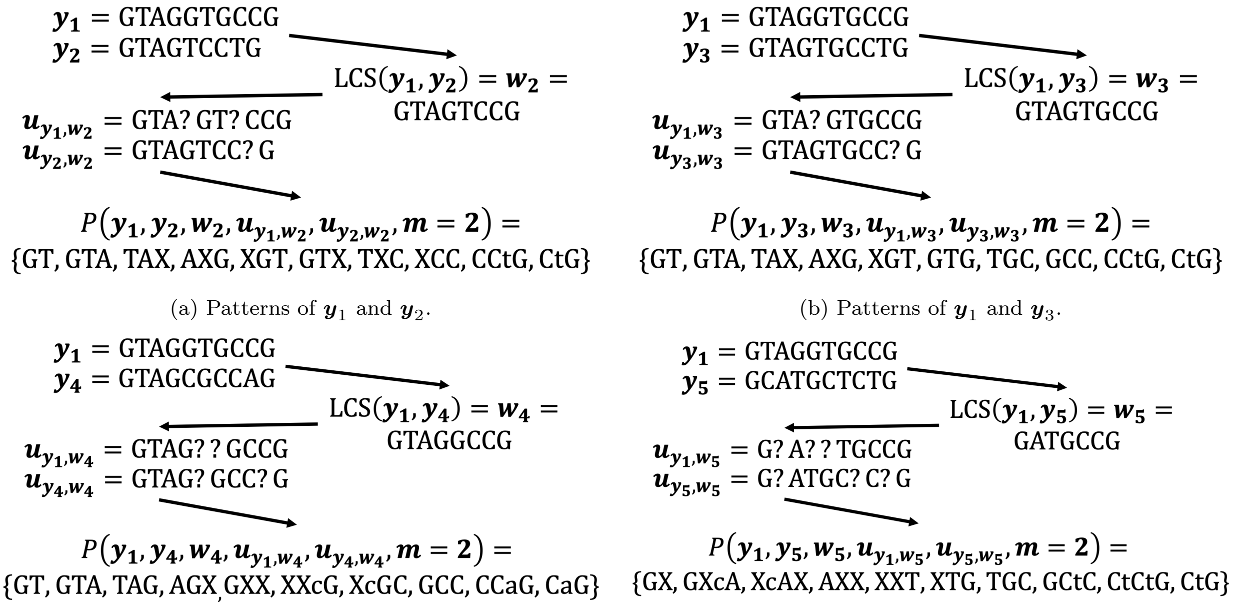

(a) Patterns of $\boldsymbol{y}_1$ and $\boldsymbol{y}_2$.

(b) Patterns of $\boldsymbol{y}_1$ and $\boldsymbol{y}_3$.

(c) Patterns of $\boldsymbol{y}_1$ and $\boldsymbol{y}_4$.

(d) Patterns of $\boldsymbol{y}_1$ and $\boldsymbol{y}_5$.

Supplementary Figure S5: Algorithm 6 Example - Patterns of $\boldsymbol{y}_1$.

2.2.3 Evaluating the patterns and their accuracy

The following list summarizes the patterns and their frequencies. Each list includes patterns from specific index. The numbers on the right side of each pattern in a list represents the pattern's frequency.

- $L[\boldsymbol{y}_1]_1 = \{\mathrm{GT}:3, \mathrm{GX}:1\}$.
- $L[\boldsymbol{y}_1]_2 = \{\mathrm{GTA}:3, \mathrm{GXcA}:1\}$.
- $L[\boldsymbol{y}_1]_3 = \{\mathrm{TAX}:2, \mathrm{TAG}:1, \mathrm{XcAX}:1\}$.
- $L[\boldsymbol{y}_1]_4 = \{\mathrm{AXG}:2, \mathrm{AGX}:1, \mathrm{AXX}:1\}$.
- $L[\boldsymbol{y}_1]_5 = \{\mathrm{XGT}:2, \mathrm{GXX}:1, \mathrm{XXT}:1\}$.
- $L[\boldsymbol{y}_1]_6 = \{\mathrm{GTG}:1, \mathrm{GTX}:1, \mathrm{XXcG}:1, \mathrm{XTG}:1\}$.
- $L[\boldsymbol{y}_1]_7 = \{\mathrm{TGC}:2, \mathrm{TXC}:1, \mathrm{XcGC}:1\}$.
- $L[\boldsymbol{y}_1]_8 = \{\mathrm{GCC}:2, \mathrm{XCC}:1, \mathrm{GCtG}:1\}$.
- $L[\boldsymbol{y}_1]_9 = \{\mathrm{CCtG}:2, \mathrm{CCaG}:1.\mathrm{CtCtG}:1\}$.
- $L[\boldsymbol{y}_1]_{10} = \{\mathrm{CtG}:3, \mathrm{CaG}:1\}$.

2.2.4 Creating the pattern path graph

Next, based on the list of patterns above, the pattern path is created. it can be shown that every pattern is a vertex in the graph, and that is represented by its sequence and its position in the sequence ($1 \leqslant i \leqslant 10$). Furthermore, the weights of the edges in the graph represents the frequencies of the patterns. As can be seen in Figure S6, the created graph is a directed acyclic graph.

2.2.5 Output

It is not hard to observe that the longest path in the pattern path graph of this example is:

$$S{\to}GT{\to}GTA{\to}TAX{\to}AXG{\to}XGT{\to}GTG\ {\to}TGC{\to}GCC{\to}CCtG{\to}CtG{\to}U,$$

and the algorithm output will be $\widehat{\boldsymbol{y}}_1 = GTAGTGCCTG = \boldsymbol{x}$.

Algorithm 5 Error Vectors Majority (EV Algorithm)

Input:

- Cluster $\mathbf{C}$ of t noisy traces: $\boldsymbol{y}_1, \boldsymbol{y}_2, \ldots, \boldsymbol{y}_t$.
- Design length $= n$
- $\boldsymbol{y}_i \in \mathbf{C}$ - a copy from the cluster.

Output:

- $\widehat{\boldsymbol{x}}$ - a revised version of $\boldsymbol{y}_i$, an estimation of $\boldsymbol{y}_i$ with less substitution and insertion errors.

1. $\mathbf{S} =$ "", an empty vector.
2. **for** $\boldsymbol{y}_k \in C$**,** $k \neq i$ **do**
   (a) Compute $EV(\boldsymbol{y}_i, \boldsymbol{y}_k)$.
   end for
3. **for** $1 \leqslant j \leqslant |\boldsymbol{y}_i| + 1$ **do**
   (a) Set $\mathbf{S}(j)$ to be the operation that appeared in the j-th entry of most of the EV that computed in Step 2.
   end for
4. Perform the operations from the vector $\mathbf{S}$ on $\boldsymbol{y}_i$ and save the resulted sequence in $\widehat{\boldsymbol{x}}$.
5. Return $\widehat{\boldsymbol{x}}$.

Algorithm 6 Pattern-Path (PP Algorithm)

Input:

- Cluster $\mathbf{C}$ of t noisy traces: $\boldsymbol{y}_1, \boldsymbol{y}_2, \ldots, \boldsymbol{y}_t$.
- Design length $= n$
- $\boldsymbol{y}_k \in \mathbf{C}$ - a copy from cluster $\mathbf{C}$.

Output:

- $\widehat{\boldsymbol{y}}_k$ - a revised version of $\boldsymbol{y}_k$. The sequence $\widehat{\boldsymbol{y}}_k$ consists of $\boldsymbol{y}_k$'s original symbols and also includes some additional symbols, which are estimations of the symbols deleted from $\boldsymbol{y}_k$.

1. $L[\boldsymbol{y}_k] = \{L[\boldsymbol{y}_k]_1, \ldots, L[\boldsymbol{y}_k]_{|\boldsymbol{y}_k|}\}$, a list of $|\boldsymbol{y}_k|$ empty lists, where each represents the list of patterns before the symbol i in $\boldsymbol{y}_i$, where the last list represents symbols before the end of the sequence.
2. /*In this stage we pair $\boldsymbol{y}_k$ with all the copies from the cluster, create list $L[\boldsymbol{y}_k]$ of $|\boldsymbol{y}_k|$ lists of patterns of symbol i and their frequencies*/
   for $\boldsymbol{y}_h \in C$ **do**
   (a) Compute $\boldsymbol{w}$ an LCS sequence of $\boldsymbol{y}_k, \boldsymbol{y}_h$.
   (b) Compute $\boldsymbol{u}_{\boldsymbol{y}_k,\boldsymbol{w}}$ an embedding sequence for $\boldsymbol{y}_k$ and $\boldsymbol{w}$.
   (c) Computes $P(\boldsymbol{y}_k, \boldsymbol{y}_h, \boldsymbol{w}, \boldsymbol{u}_{\boldsymbol{y}_k,\boldsymbol{w}}, m = 3)$.
   (d) For each $1 \leqslant i \leqslant \boldsymbol{y}_k$ add to $L[\boldsymbol{y}_h]_i$ the pattern $P(\boldsymbol{y}_k, \boldsymbol{y}_h, \boldsymbol{w}, \boldsymbol{u}_{\boldsymbol{y}_k,\boldsymbol{w}}, i, 3)$.
   end for
3. Build $G_{pat} = (V, E)$ - the pattern graph.
4. Find the longest path from the source vertex S in G_{pat}.
5. Let $\widehat{\boldsymbol{y}}_k$ bet the sequence that inherited from the patterns of the vertices of the longest path.
6. Return $\widehat{\boldsymbol{y}}_k$.

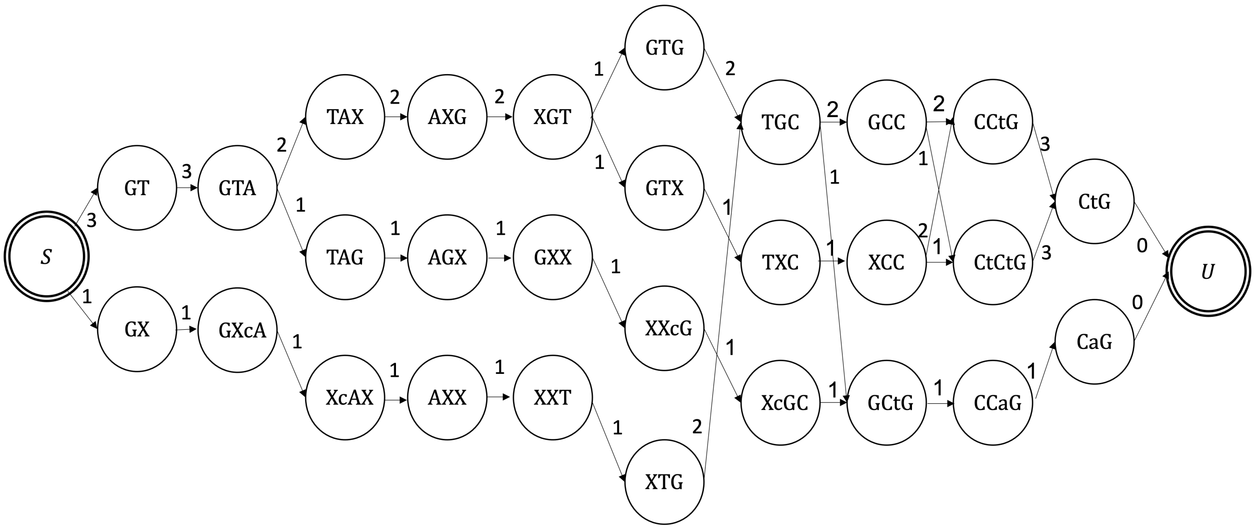

Supplementary Figure S6: The pattern-path graph.

3 Results

3.1 Results on Simulated Data

We evaluated the accuracy of Algorithm 4 by simulations. First, we recall our interpretation of the deletion-insertion-substitution channel. In our deletion-insertion-substitution channel, the sequence is transmitted symbol-by-symbol. First, before transmitting the symbol, it checks for an insertion error before the transmitted symbol. The channel flips a coin, and in probability p_i, an insertion error occurs before the transmitted symbol. If an insertion error occurs, the inserted symbol is chosen uniformly. Then, the channel checks for a deletion error, and again flips a coin, and in probability p_d the transmitted symbol is deleted. Lastly, the channel checks for a substitution error. The channel flips a coin, and in probability p_s the transmitted symbol is substituted to another symbol. The substituted symbol is chosen uniformly. In case that both deletion and substitution errors occurs in the same symbol, we refer to it as a substitution.

We simulated 100,000 clusters of sizes $t = 10, 20$, the sequences length was $n = 100$, and the alphabet size was $q = 4$. The deletion, insertion, and substitution probabilities were all identical, and ranged between 0.01 and 0.1. It means that the actual error probability of each cluster was $1 - (1 - p_i)(1 - p_s)(1 - p_d)$ and ranged between 0.029701 and 0.271. We reconstructed the original sequences of the clusters using Algorithm 4 and the algorithms from [4] and from [11]. For each algorithm we evaluated its edit error rate, the success rate, and the value of k_{1_succ}. The edit error rates of Algorithm 4 were the lowest among the tested algorithms, while the algorithm from [11] presented the highest edit error rates. Moreover, it can be seen that our algorithms presented significantly low edit error rates value for higher values of error probabilities. In addition, they also presented the lowest value of k_{1_succ}. For example, when the cluster size was $t = 20$ and the error probability was $p = 0.142625$, the value of k_{1_succ} of Algorithm 4 was 2, while the other algorithms presented k_{1_succ} values of at least 12. The results of these simulations for cluster sizes of $t = 10$ and $t = 20$ can be found in Figure S7, Figure S8 and Figure S9.

4 Conclusions

We presented in this paper several new algorithms for the deletion DNA reconstruction problem and for the DNA reconstruction problem. While most of the previously published algorithms use a symbol-wise majority approaches, our algorithms look globally on the entire sequence of the traces, and use the LCS or SCS of a given set of traces. Our algorithms are designed to specifically support DNA storage systems and to reduce the edit error rate of the reconstructed sequences. According to our tests on simulated data and on data from DNA storage experiments, we found out that our algorithms significantly reduced the error rates compared to the previously published algorithms. Moreover, our algorithms performed even better when the error probabilities were high, while using less traces than the other algorithms. Even though our algorithms improved previous results, there are still several challenges that need to be addressed in order to

Algorithm 7 Iterative Reconstruction - Horizontal (The HR Algorithm)

Input:

- Cluster **C** of t noisy traces: $\boldsymbol{y}_1, \boldsymbol{y}_2, \ldots, \boldsymbol{y}_t$.
- Design length $= n$.

Output:

- $\mathbf{S}$=$\{\boldsymbol{s}_1, \boldsymbol{s}_2, \ldots, \boldsymbol{s}_p\}$, a multiset of p candidates, that estimate the original sequence of the cluster.

1. $\mathbf{S} = \emptyset$, $\mathbf{C}_{orig} = \mathbf{C}$
2. **for** j= 1, 2, ..., k **do**
   (a) $\mathbf{C}_{tmp} = \emptyset$
   (b) **for** $\boldsymbol{y}_i \in \mathbf{C}_{orig}$ **do**
      i. Perform Algorithm 5 on $\boldsymbol{y}_i$ to correct substitution errors.
      ii. Perform Algorithm 6 on $\boldsymbol{y}_i$ to correct deletion errors.
      iii. Perform Algorithm 5 on $\boldsymbol{y}_i$ to correct insertion errors.
      iv. $\mathbf{C}_{tmp} = \mathbf{C}_{tmp} \cup \{\boldsymbol{y}_i\}$.
   (c) **end for**
   (d) $\mathbf{C} = \mathbf{C}_{tmp}$.
   end for
3. $\mathbf{S} = \mathbf{S} \cup \mathbf{C}$.
4. Set $\mathbf{C}_{orig} = \mathbf{C}^R_{orig}$ and repeat Steps 2-3 on $\mathbf{C}^R_{orig}$. Add the results to $\mathbf{S}$.

fully solve the DNA reconstruction problem. Some of these challenges are listed as follows.

1. Design efficient reconstruction algorithms that improve the current edit error rate.
2. Design error correcting codes for DNA storage systems.
3. Design efficient coded trace reconstruction algorithms for DNA storage systems.
4. Standardization of reconstruction algorithms for DNA storage systems.

Acknowledgment

The authors of this paper thank Prof. Roee Amit, Dr. Sarah Goldberg and Dr. Nanami Kikuchi for their help with the PCR process of the published libraries, and for the fruitful discussion and lab equipment. The author also wish to thank the Technion Genome Center, specifically Liat Linde and Nitsan Fourier, for their help with the sequencing process of the libraries. The authors thank Matika Lidgi for her help with the divider BMA algorithm and Rotem Samuel for his help with the implementations and simulations of the algorithms in the paper. They also thank Cyrus Rashtchian for helpful discussions. We thank Prof. Gala Yadgar for her invaluable help with the simulation infrastructure. Finally, we thank Daniella Bar-Lev for helpful and inspiring discussions along the way.

References

[1] A. Atashpendar, M. Beunardeau, A. Connolly, R. Géraud, D. Mestel, A. W. Roscoe, and P. Y. A. Ryan. From clustering supersequences to entropy minimizing subsequences for single and double deletions. *CoRR*, abs/1802.00703, 2018.

Algorithm 8 Iterative Reconstruction - Vertical (The VR Algorithm)

Input:

- Cluster $\mathbf{C}$ of t noisy traces: $\boldsymbol{y}_1, \boldsymbol{y}_2, \ldots, \boldsymbol{y}_t$.
- Design length $= n$.

Output:

- $\mathbf{S}=\{\boldsymbol{s}_1, \boldsymbol{s}_2, \ldots, \boldsymbol{s}_p\}$, a multiset of p candidates, sequences that estimates the original sequence of the cluster.

1. $\mathbf{S} = \emptyset$, $\mathbf{C}_{orig} = \mathbf{C}$
2. **for** j= 1, 2, ..., k **do**
   (a) $\mathbf{C}_{tmp} = \emptyset$
   (b) **for** $\boldsymbol{y}_i \in \mathbf{C}$ **do**
      i. Perform Algorithm 5 on $\boldsymbol{y}_i$ to correct substitution errors.
      ii. $\mathbf{C}_{tmp} = \mathbf{C}_{tmp} \cup \{\boldsymbol{y}_i\}$.
   (c) **end for**
   (d) $\mathbf{C} = \mathbf{C}_{tmp}$
   (e) $\mathbf{C}_{tmp} = \emptyset$
   (f) **for** $\boldsymbol{y}_i \in \mathbf{C}$ **do**
      i. Perform Algorithm 6 on $\boldsymbol{y}_i$ to correct deletion errors.
      ii. $\mathbf{C}_{tmp} = \mathbf{C}_{tmp} \cup \{\boldsymbol{y}_i\}$.
   (g) **end for**
   (h) $\mathbf{C} = \mathbf{C}_{tmp}$
   (i) $\mathbf{C}_{tmp} = \emptyset$
   (j) **for** $\boldsymbol{y}_i \in \mathbf{C}$ **do**
      i. Perform Algorithm 5 on $\boldsymbol{y}_i$ to correct insertion errors.
      ii. $\mathbf{C}_{tmp} = \mathbf{C}_{tmp} \cup \{\boldsymbol{y}_i\}$.
   (k) **end for**
   (l) $\mathbf{C} = \mathbf{C}_{tmp}$
   end for
3. $\mathbf{S} = \mathbf{S} \cup \mathbf{C}$
4. Set $\mathbf{C}_{orig} = \mathbf{C}^R_{orig}$ and repeat Steps 2-3 on $\mathbf{C}^R_{orig}$. Add the results to $\mathbf{S}$.

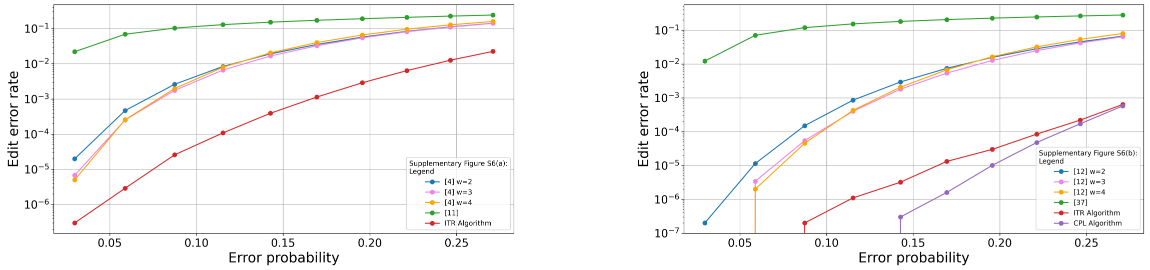

(a) Edit error rate by the error probability for $t = 10$. The X-axis represents the error probability of the simulated clusters and the Y-axis represents the edit error rate.

(b) Edit error rate by the error probability for $t = 20$. The X-axis represents the error probability of the simulated clusters and the Y-axis represents the edit error rate.

Supplementary Figure S7: Edit error rate by the error probabilities for the cluster sizes $t = 10$ and $t = 20$. The length of the original sequence was $n = 100$ and the error probabilities ranges between 0.029701 and 0.271.

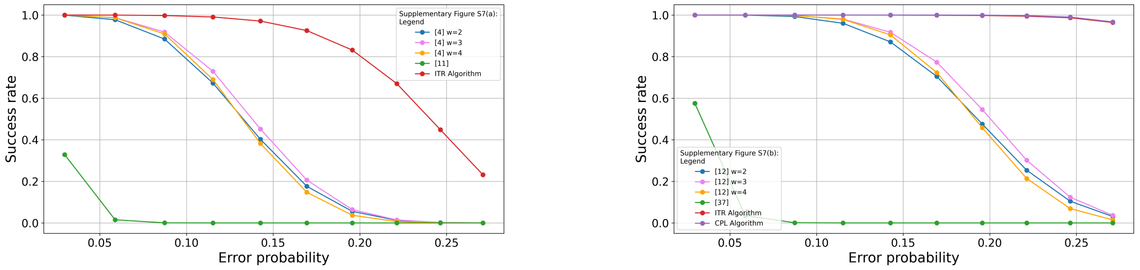

(a) Success rate by the error probability for $t = 10$. The X-axis represents the error probability of the simulated clusters and the Y-axis represents the success rate.

(b) Success rate by the error probability for $t = 20$. The X-axis represents the error probability of the simulated clusters and the Y-axis represents the success rate.

Supplementary Figure S8: Success rate by the error probabilities for the cluster sizes $t = 10$ and $t = 20$. The length of the original sequence was $n = 100$ and the error probabilities ranges between 0.029701 and 0.271.

[2] T. Batu, S. Kannan, S. Khanna, and A. McGregor. Reconstructing strings from random traces. In *Proceedings of the fifteenth annual ACM-SIAM symposium on Discrete algorithms*, pages 910–918. Society for Industrial and Applied Mathematics, 2004.

[3] C. Elzinga, S. Rahmann, and H. Wang. Algorithms for subsequence combinatorics. *Theoretical Computer Science*, 409(3):394–404, 2008.

[4] P. S. Gopalan, S. Yekhanin, S. D. Ang, N. Jojic, M. Racz, K. Strauss, and L. Ceze. Trace reconstruction from noisy polynucleotide sequencer reads, July 26 2018. US Patent App. 15/536,115.

[5] S. Y. Itoga. The string merging problem. *BIT Numerical Mathematics*, 21(1):20–30, 1981.

[6] L. Organick, S. D. Ang, Y.-J. Chen, R. Lopez, S. Yekhanin, K. Makarychev, M. Z. Racz, G. Kamath, P. Gopalan, B. Nguyen, C. N. Takahashi, S. Newman, H.-Y. Parker, C. Rashtchian, K. Stewart, G. Gupta, R. Carlson, J. Mulligan, D. Carmean, G. Seelig, L. Ceze, and K. Strauss. Random access in large-scale DNA data storage. *Nature Biotechnology*, 36:242 EP –, 02 2018.

[7] O. Sabary, Y. Orlev, R. Shafir, L. Anavy, E. Yaakobi, and Z. Yakhini. Solqc: Synthetic oligo library quality control tool. *BioRxiv*, page 840231, 2019.

[8] O. Sabary, E. Yaakobi, and A. Yucovich. The error probability of maximum-likelihood decoding over two deletion channels. *arXiv preprint arXiv:2001.05582*, 2020.

[9] S. R. Srinivasavaradhan, M. Du, S. Diggavi, and C. Fragouli. On maximum likelihood reconstruction over multiple deletion channels. In *2018 IEEE International Symposium on Information Theory (ISIT)*, pages 436–440. IEEE, 2018.

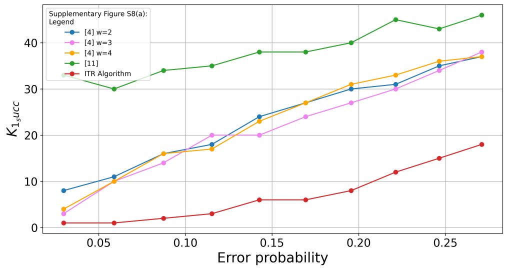

(a) k_{1_succ} values by the error probability for $t = 10$. The X-axis represents the error probability and the Y-axis represents the value of k_{1_succ}.

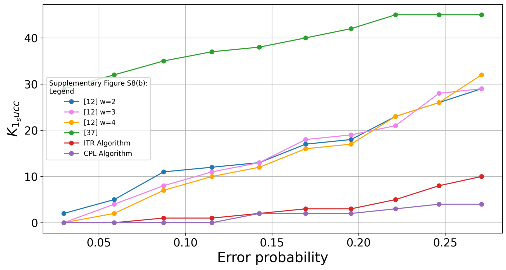

(b) k_{1_succ} values by the error probability for $t = 20$. The X-axis represents the error probability and the Y-axis represents the value of k_{1_succ}.

Supplementary Figure S9: k_{1_succ} values by the error probabilities for the cluster sizes $t = 10$ and $t = 20$. The length of the original sequence was $n = 100$ and the error probabilities ranges between 0.029701 and 0.271.

[10] S. R. Srinivasavaradhan, S. Gopi, H. D. Pfister, and S. Yekhanin, Trellis BMA: Coded trace reconstruction on IDS channels for DNA storage. *IEEE International Symposium on Information Theory (ISIT)*, pages 2453–2458, 2021.

[11] K. Viswanathan and R. Swaminathan. Improved string reconstruction over insertion-deletion channels. In *Proceedings of the nineteenth annual ACM-SIAM symposium on Discrete algorithms*, pages 399–408, 2008.
